# Supplementary figures and images for: Drainage-structuring of ancestral variation and a common functional pathway shape limited genomic convergence in natural high- and low-predation guppies
Source: PLoS Genet. 2021 May 24;17(5):e1009566. doi: 10.1371/journal.pgen.1009566 (PMC8177651; doi:10.1371/journal.pgen.1009566)

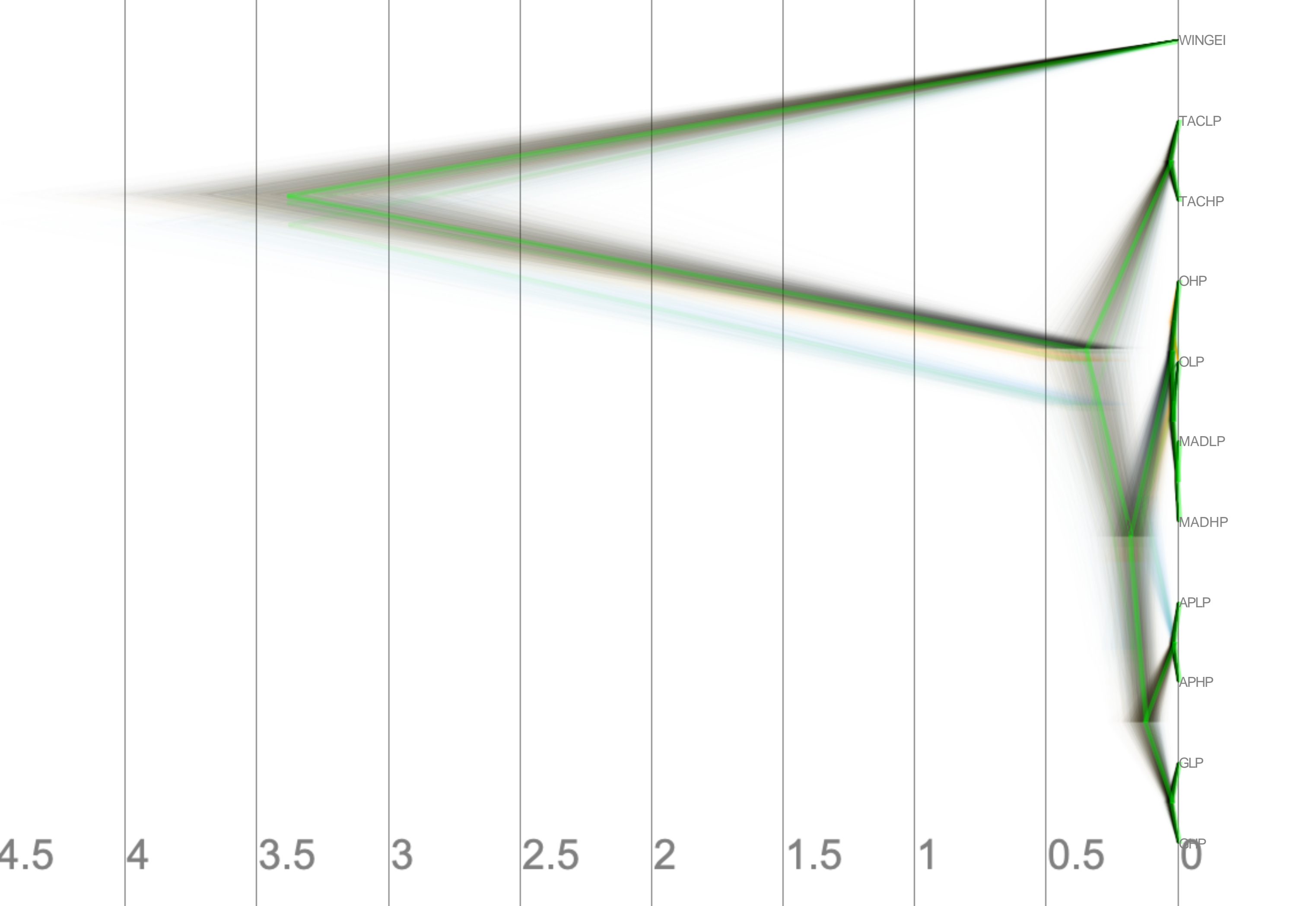

Supplement: S1 Fig — Grid lines denote divergence times in millions of years (mya). Black trees are those that share the most common topology. Orange trees were the second most common topology, and blue the third. The consensus tree is marked as a solid green tree. Trees plotted are based on merged post-burn-in trees sampled every 1000 iterations from a total of 2,200,000 MCMC iterations. (PDF) [file pgen.1009566.s001.pdf]

### Aripo removed

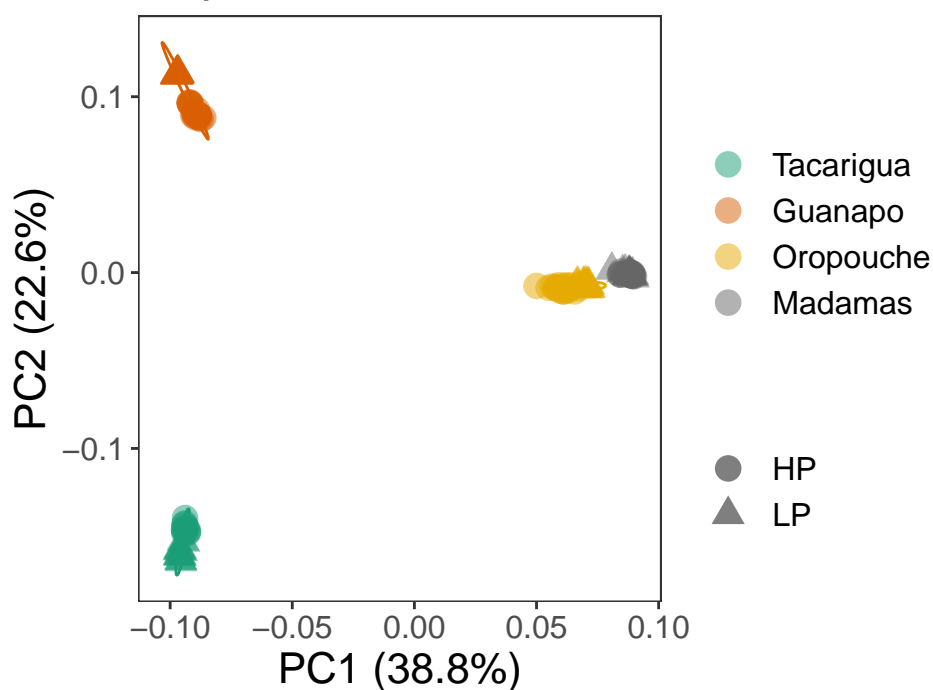

### Guanapo removed

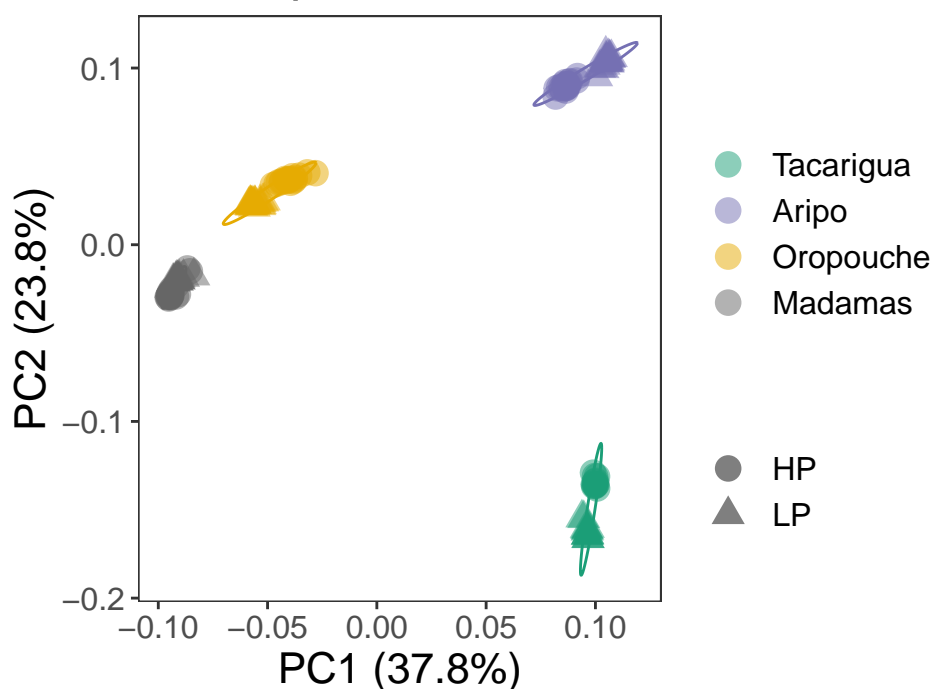

### Tacarigua removed

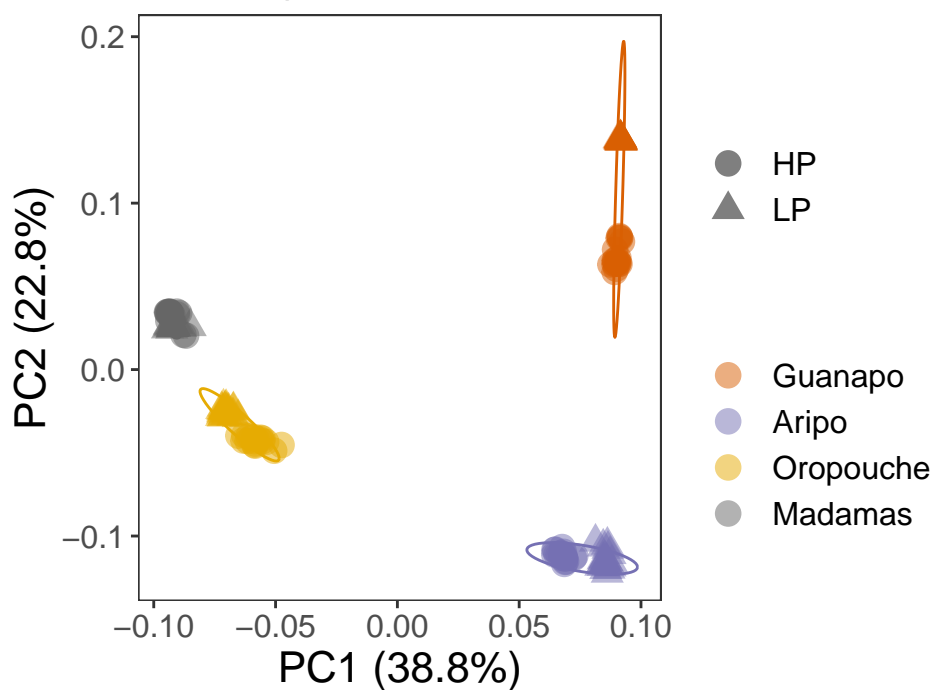

Supplement: S2 Fig — These results demonstrate that our population structure results are unlikely to be driven a sample size bias towards Caroni rivers. Each panel shows PC1 and PC2 with % variance explained. Point colour and shape reflect river and predation environment respectively. Ellipses show 95% confidence around river groups. In all cases, PC1 reflects the split between Caroni and non-Caroni populations, and PC2 shows structure within the Caroni drainage. (PDF) [file pgen.1009566.s002.pdf]

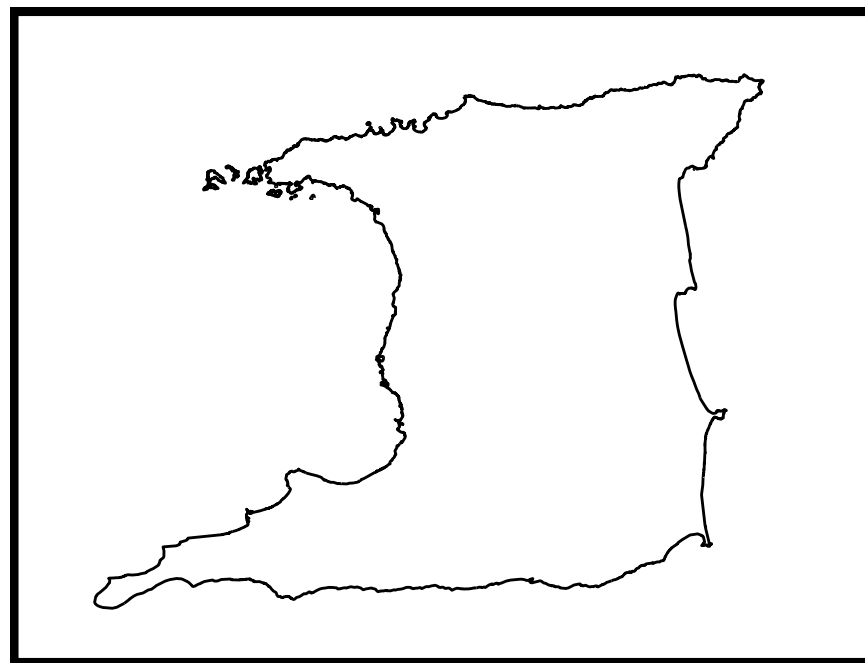

River

- Tacarigua
- Guanapo
- Aripo
- Oropouche
- Madamas

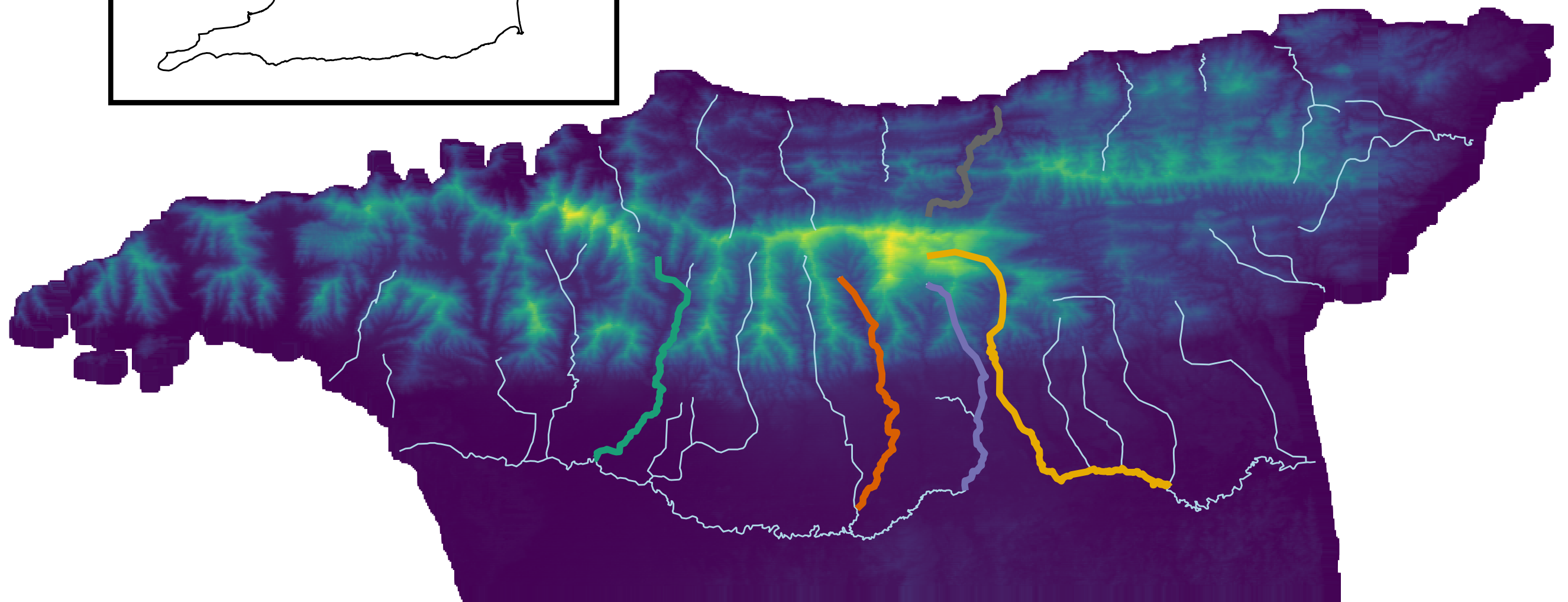

Supplement: S3 Fig — Sampling rivers are coloured according to legend and other major rivers are coloured light blue. Topography ranges from low altitude (dark fill) to high altitude (light fill). Upstream regions of rivers in the western Caroni drainage (Tacarigua, Guanapo and Aripo) are flanked by mountain ranges, likely preventing gene flow occurring between these rivers. The coastline shapefile was sourced from OpenStreetMap (openstreetmap.org; CC BY-SA 2.0), rivers were added manually and are available as shapefiles at Zenodo doi: 10.5281/zenodo.4740381, and elevation rasters were sourced from the US Geological Survey (earthexplorer.usgs.gov; SRTM; US Public Domain). (PDF) [file pgen.1009566.s003.pdf]

GLP

APLP

OLP

MADLP

TACLP

GLP

APLP

OLP

Freq  
(log)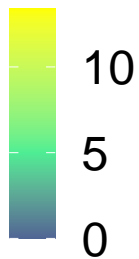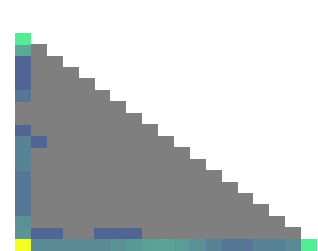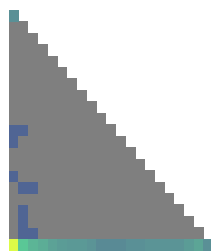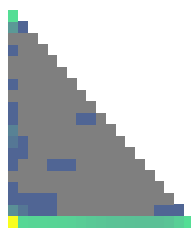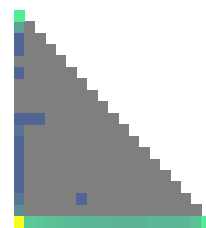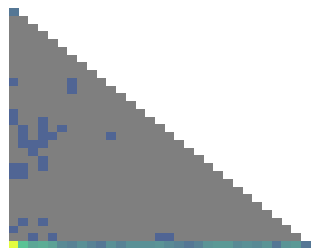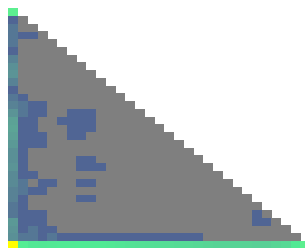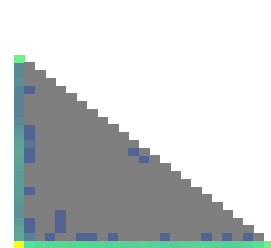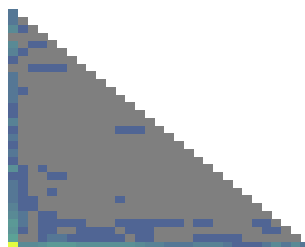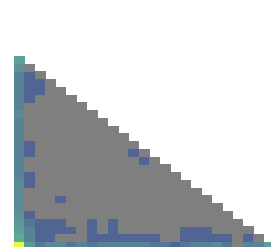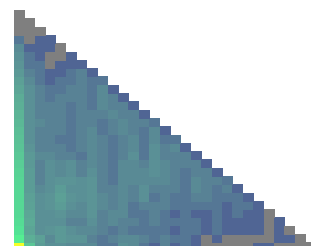

Supplement: S4 Fig — This figure demonstrates excesses of private variation as densities limited to the first column or first row. Beyond these regions of the sfs, genetic variation is typically limited to regions close to the first row or column, representing sites that are at low frequency in either population. These signatures are emblematic of minimal shared genetic variation among these populations. The exception to this pattern is MADLP-OLP, where the excess of OLP private alleles in the first column suggests gene flow in direction of MADLP > OLP. In each sfs, the frequency of sites in each population is illustrated from 0 to 2 N, where N is the number of individuals in each population. Projections have been scaled so each LP population has the same value of N (which distorts the shape of SFS across rows/columns. Each cell within these 2dsfs therefore shows the density (log-transformed) of SNPs with relevant allele counts in each population. Cells within the first column and first row show private alleles that are absent in one population (allele count of 0). Grey cells are missing data, where no SNPs are found at allele counts of x and y in LP and HP populations respectively. (PDF) [file pgen.1009566.s004.pdf]

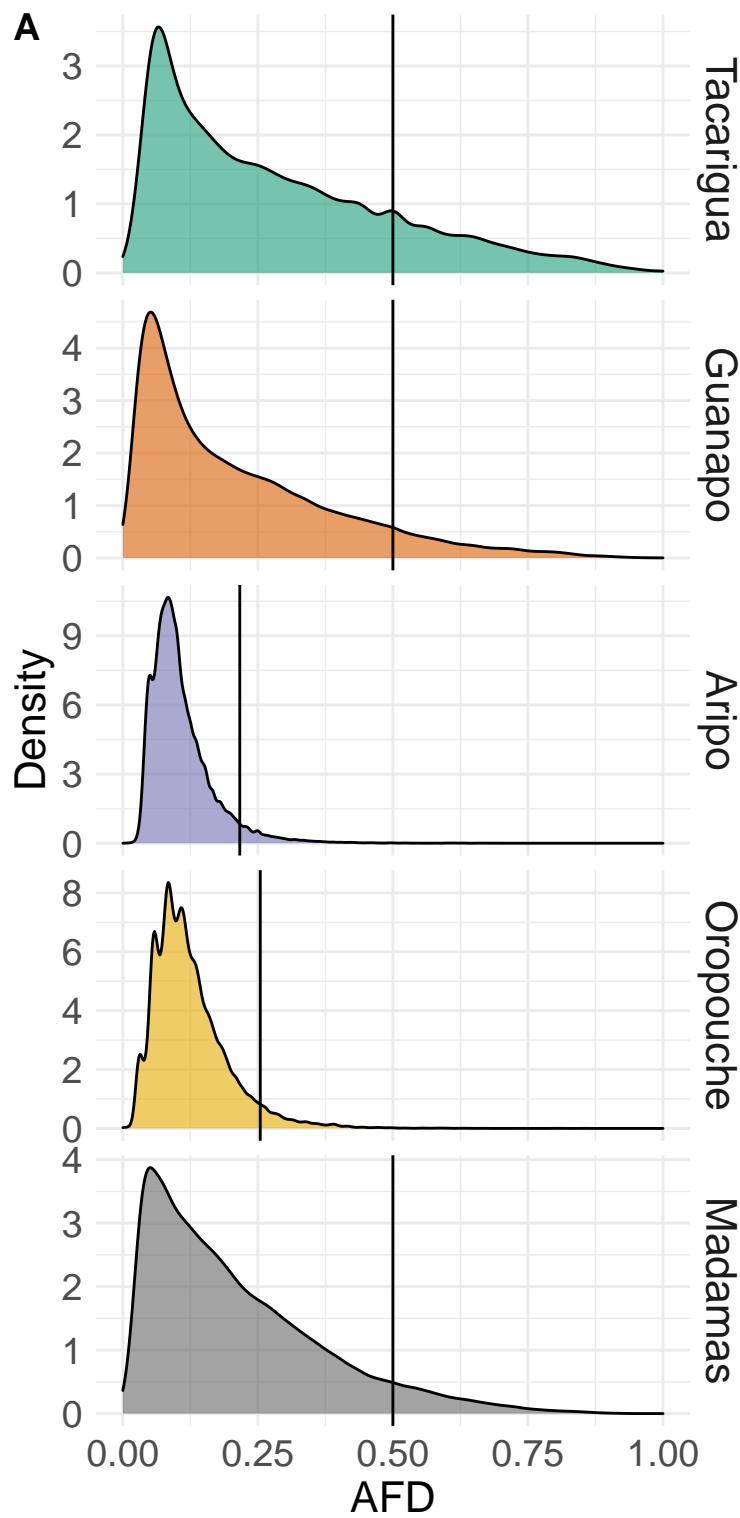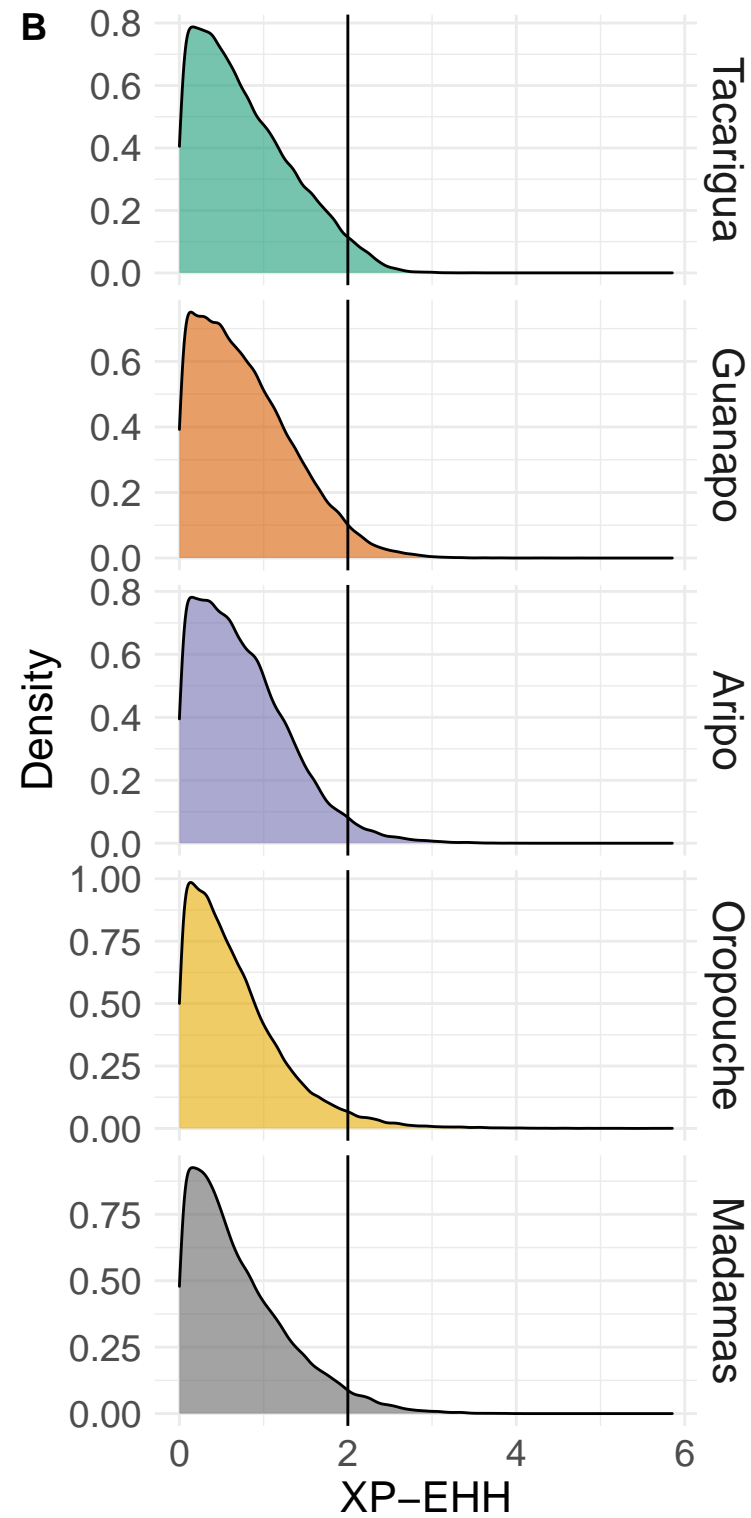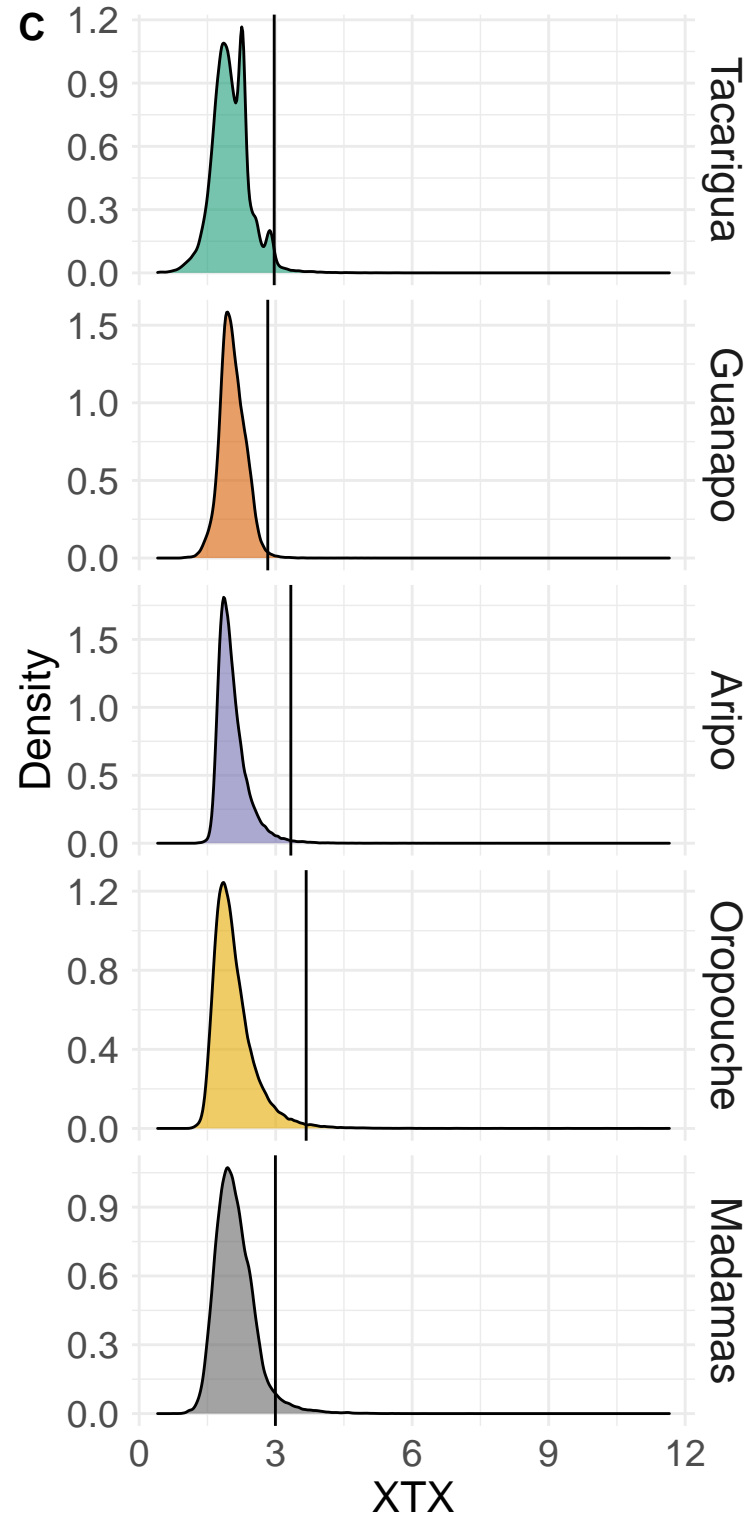

Supplement: S5 Fig — Distributions of selection scanning methods within each river and their associated outlier cut-offs for AFD (A), XP-EHH (B) and XtX (C). (PDF) [file pgen.1009566.s005.pdf]

**A** Overlapping Selection Scan Outlier Windows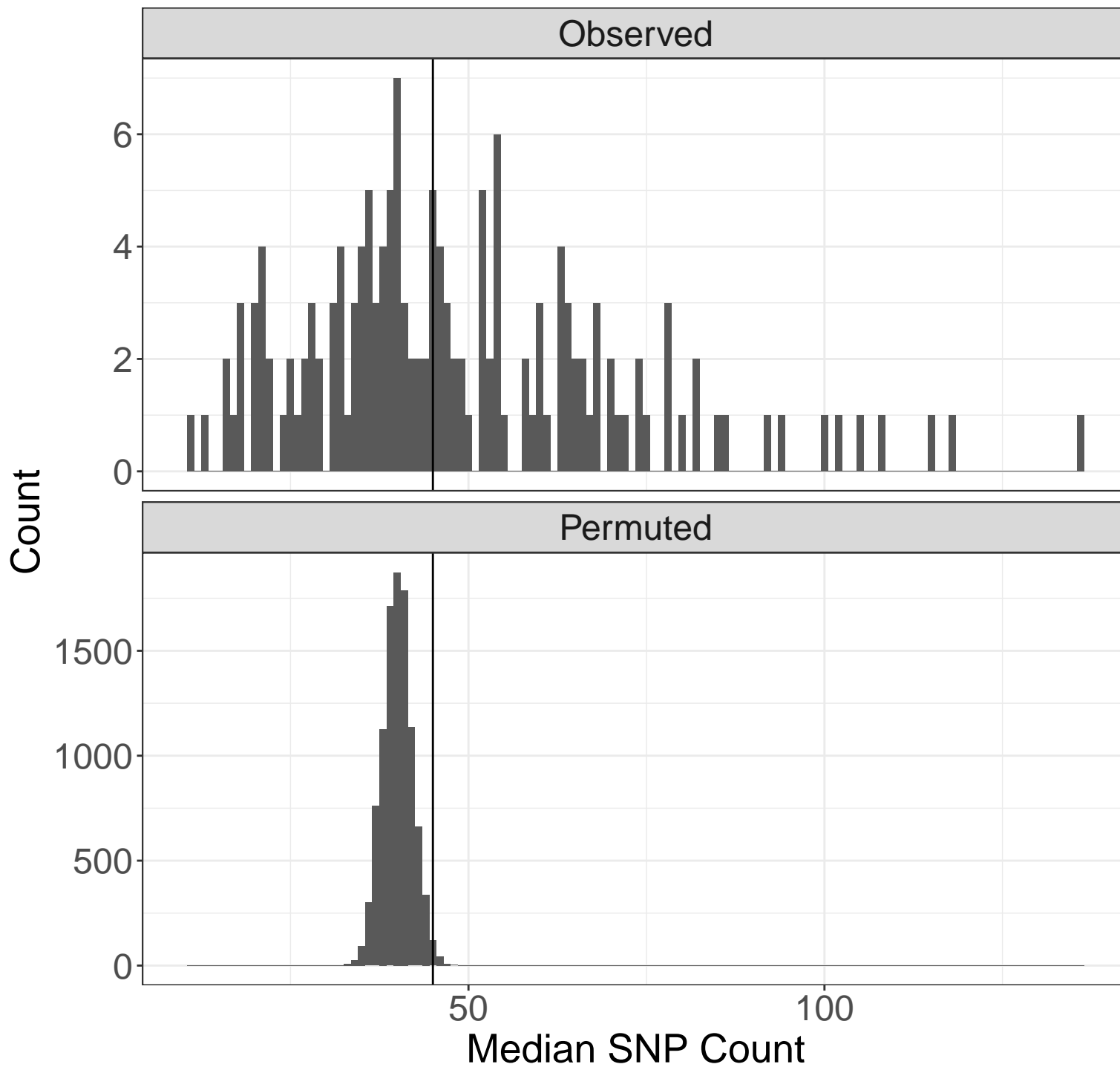**B** Baypass Outlier Windows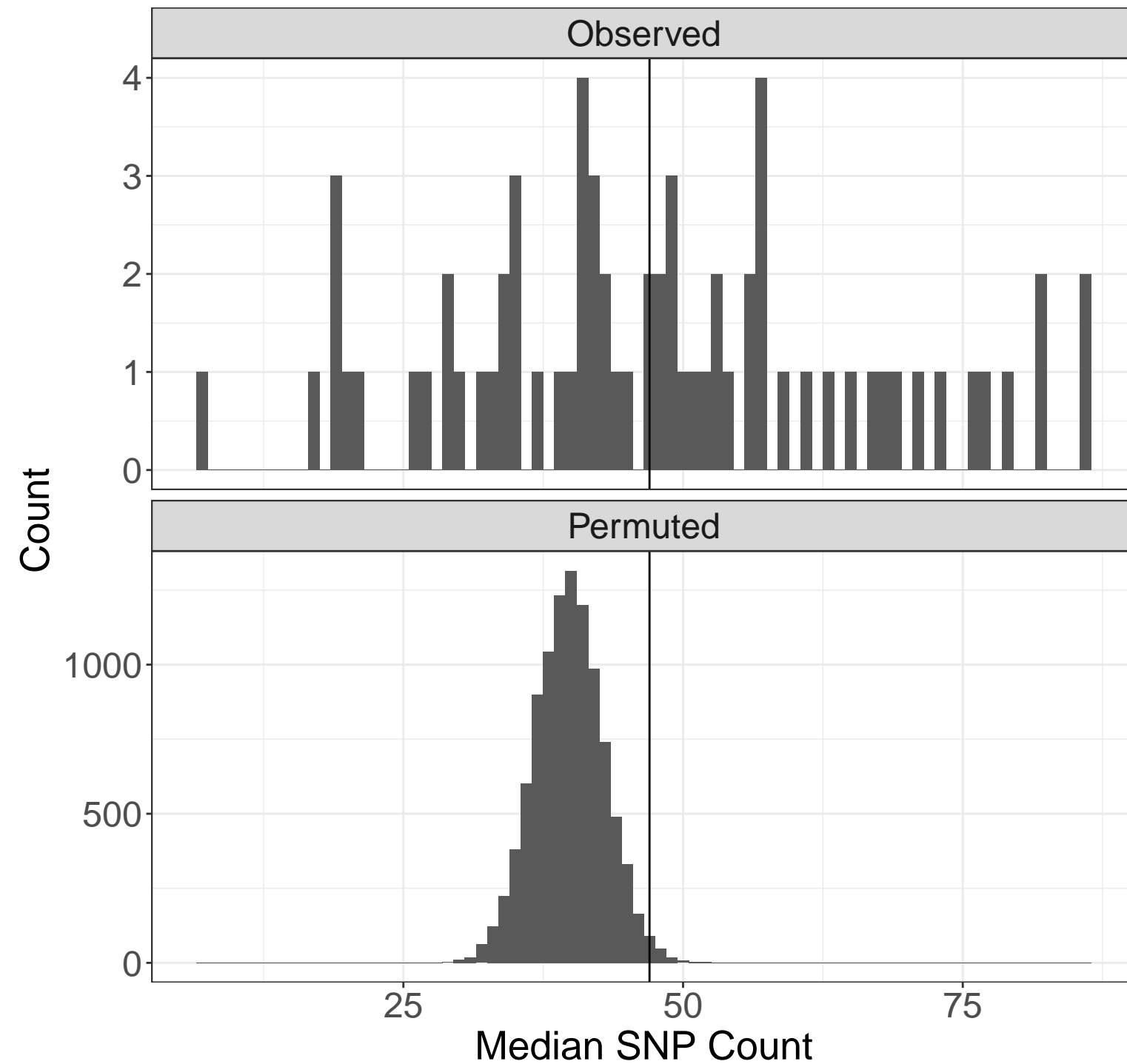

Supplement: S6 Fig — SNP counts within all overlapping selection scan outlier windows (A) (within-river outlier for two or more selection scans in >1 river) and BayPass outlier windows (B) (>99.9% quantile). The first row shows the distribution of observed SNP counts within outlier windows. The second row shows the permuted distribution of median SNP counts for 10,000 randomly drawn window sets from the total genome set, each of which contains N windows, where N = the number of observed outlier windows in the first row. The median SNP count of the observed outlier windows is shown in each panel as a solid vertical line. Under a one-tailed hypothesis, respective permuted p-values were 0.0172 (A) and 0.0176 (B). (PDF) [file pgen.1009566.s006.pdf]

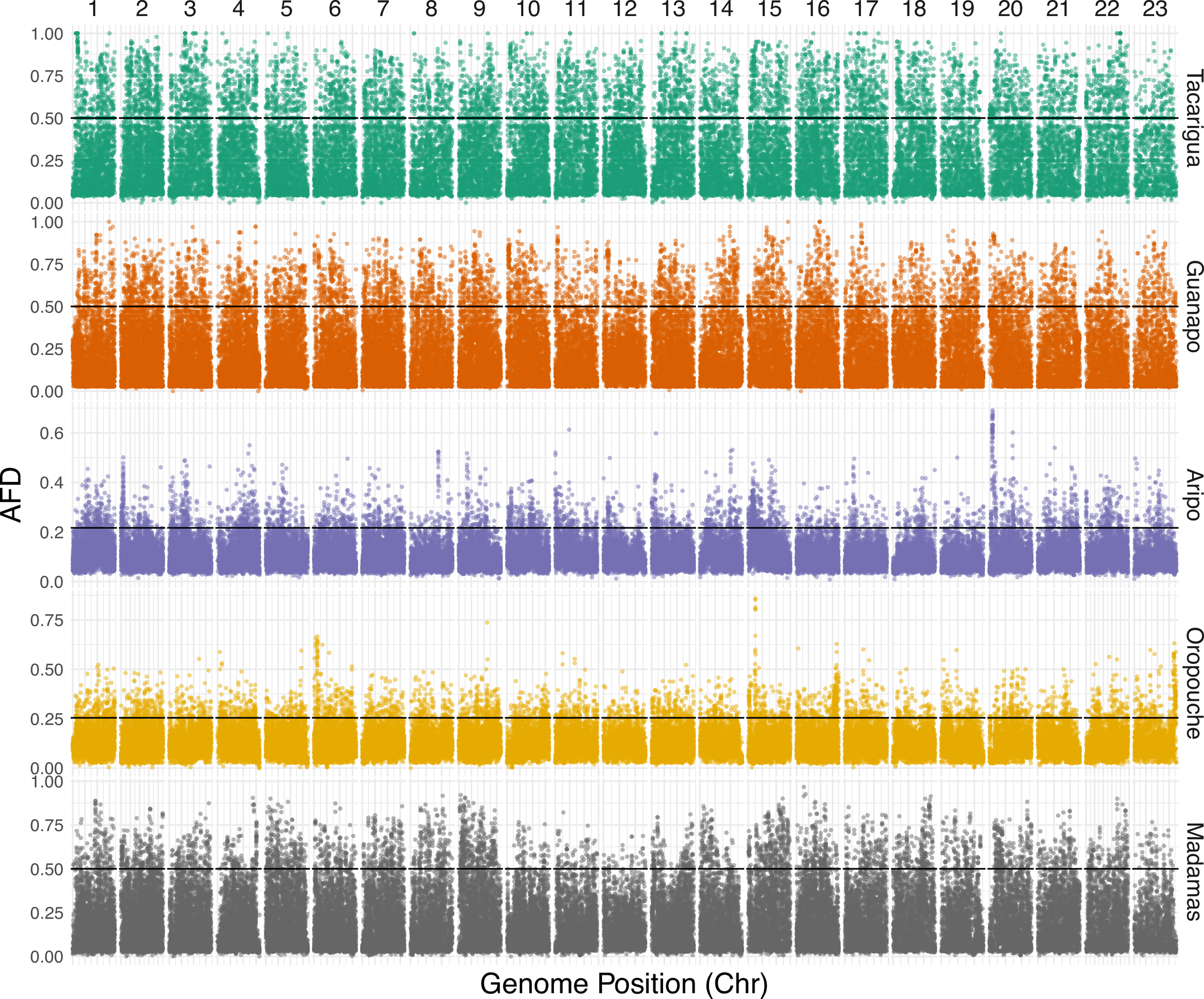

Supplement: S7 Fig — Panels represent the 23 chromosomes in the guppy genome. Each row represents the change in the absolute allele frequency for 10kb windows between HP and LP populations in a different river. Chr20 has been updated to include the unplaced scaffold 000094F. The horizontal line in each row denotes river-specific outlier cutoffs. (TIF) [file pgen.1009566.s007.tif]

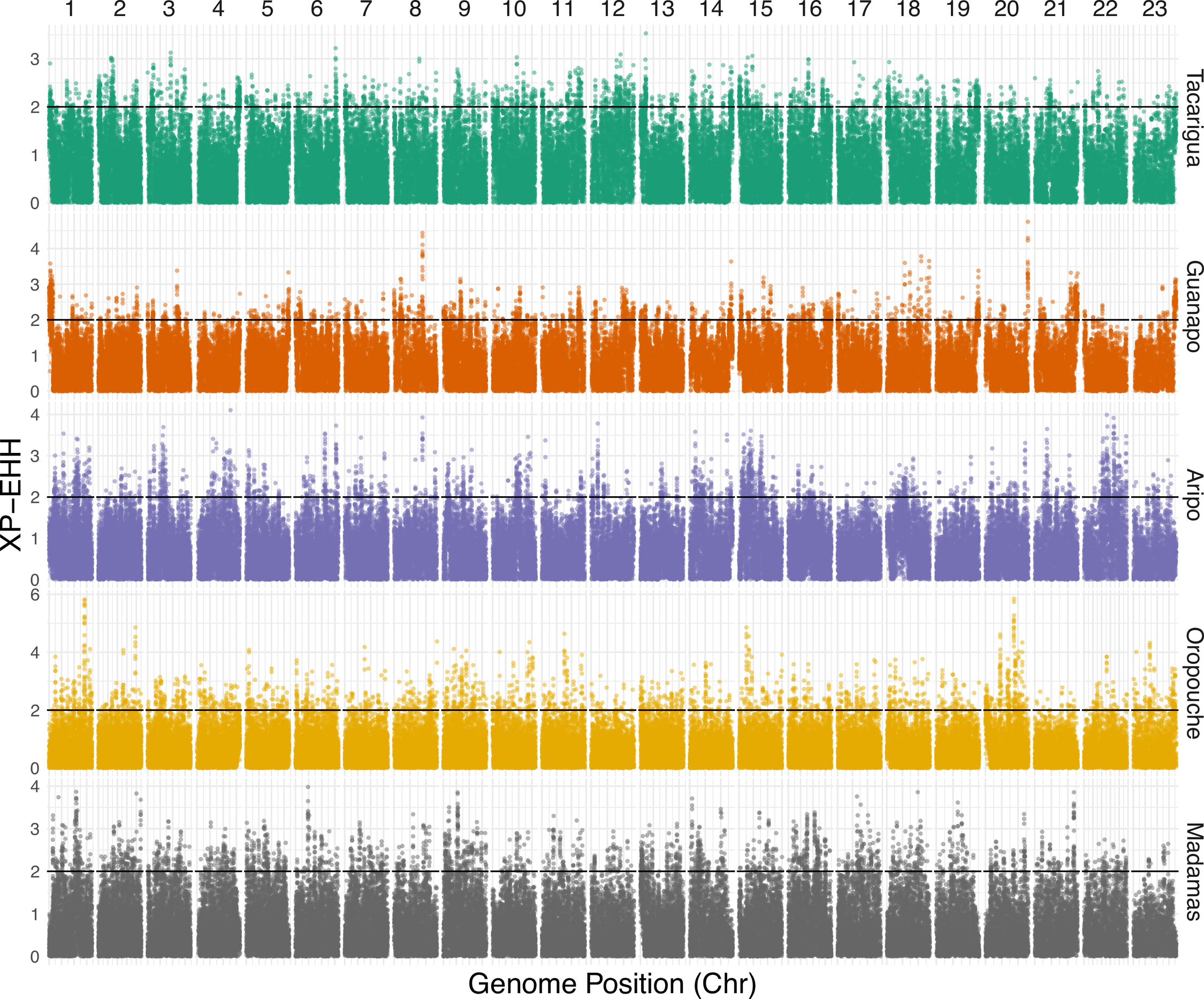

Supplement: S8 Fig — Panels represent the 23 chromosomes in the guppy genome. Each row represents the normalised score for XP-EHH, which compares extended haplotype homozygosity between HP and LP populations within rivers (absolute-transformed). Chr20 has been updated to include the unplaced scaffold 000094F. The horizontal line in each row denotes the outlier cutoff = 2, analogous to a Z-score > 2 reflecting approximately p = 0.05 following normalisation. (TIF) [file pgen.1009566.s008.tif]

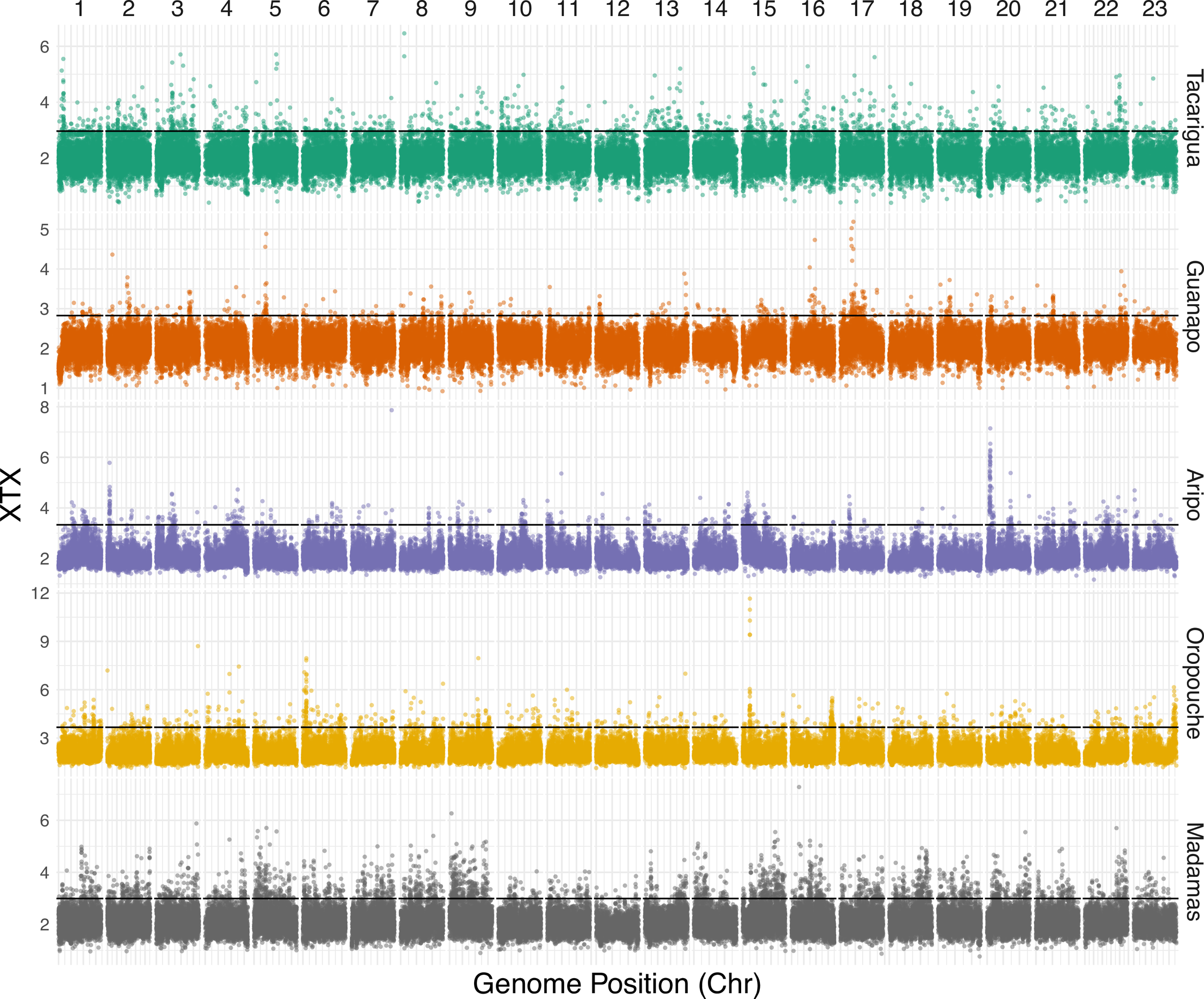

Supplement: S9 Fig — Panels represent the 23 chromosomes in the guppy genome. Each row represents the XtX score (a Bayesian analogue of FST, describing relative genetic differentiation) for 10kb windows between HP and LP populations in a different river. Chr20 has been updated to include the unplaced scaffold 000094F. The horizontal line in each row denotes river-specific outlier cutoffs, calculated according to neutral simulations of XtX within each river. (TIF) [file pgen.1009566.s009.tif]

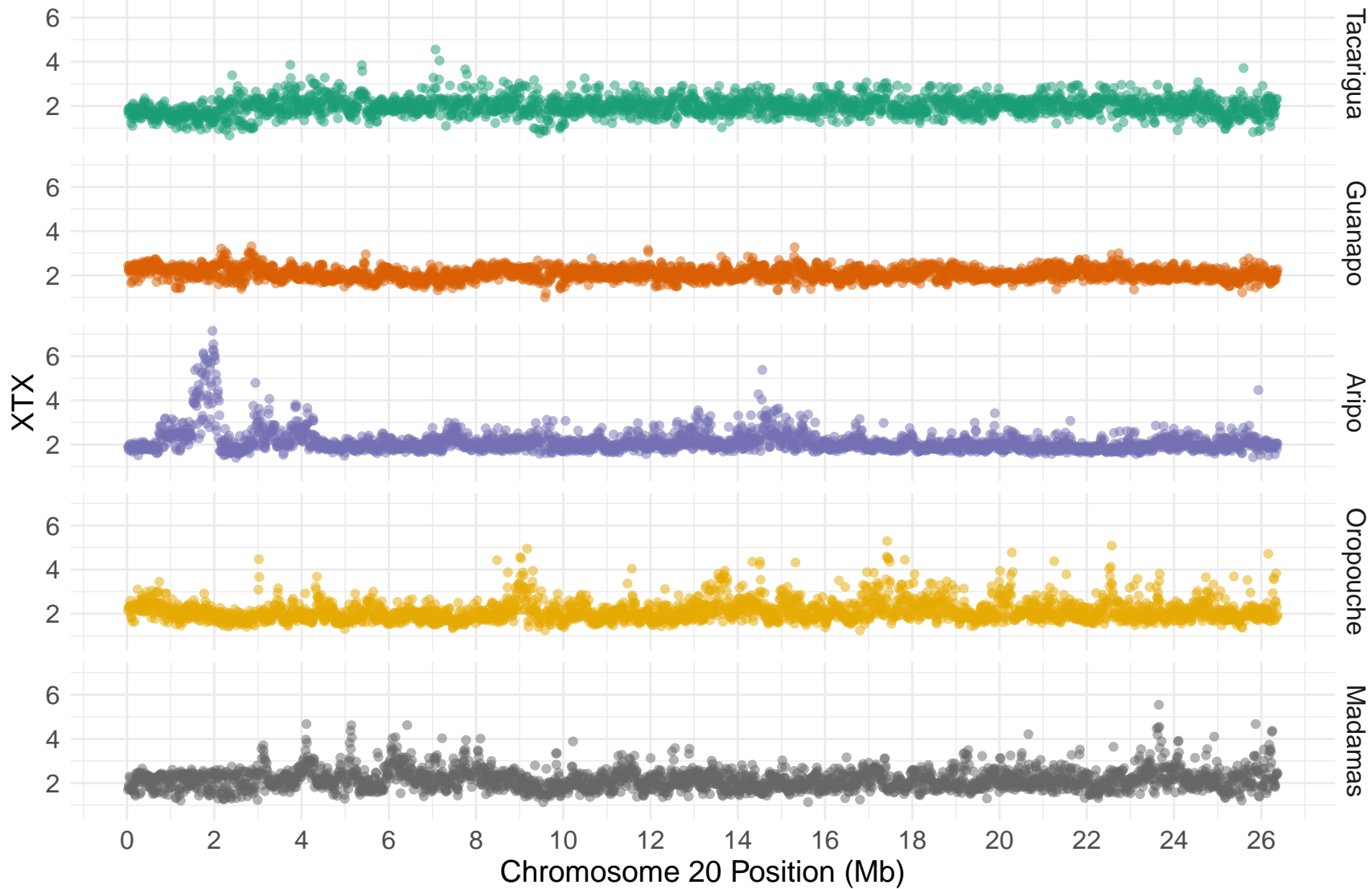

Supplement: S11 Fig — Each row represents the XtX score (a Bayesian analogue of FST, describing relative genetic differentiation) for 10kb windows between HP and LP populations in a different river. Chr20 has been updated to include the unplaced scaffold 000094F. This figure highlights the location of a peak of strong HP-LP differentiation int the Aripo river. (PDF) [file pgen.1009566.s011.pdf]

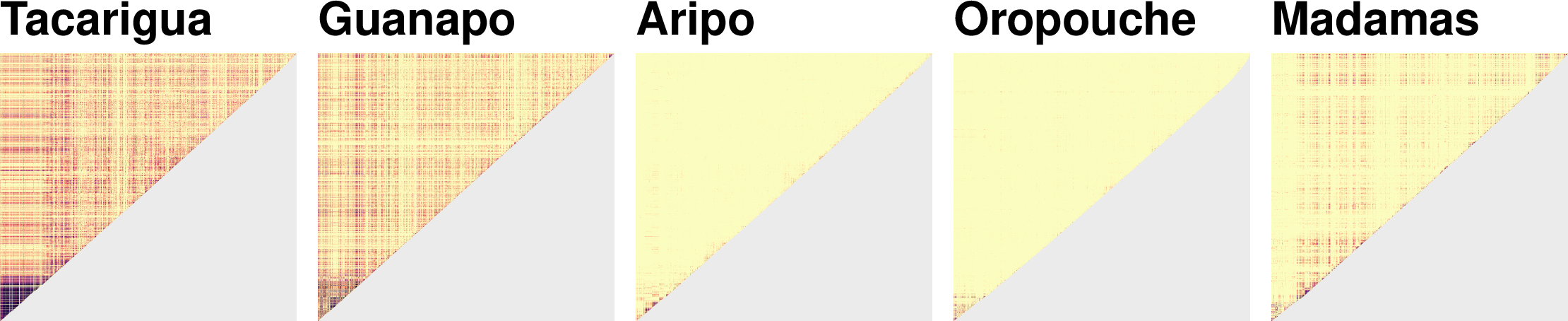

Supplement: S12 Fig — Dark regions highlight elevated linkage (R2), and light regions are low linkage. These plots highlight elevated linkage disequilibrium among SNPs at the start of chromosome 20 in several rivers. (TIF) [file pgen.1009566.s012.tif]

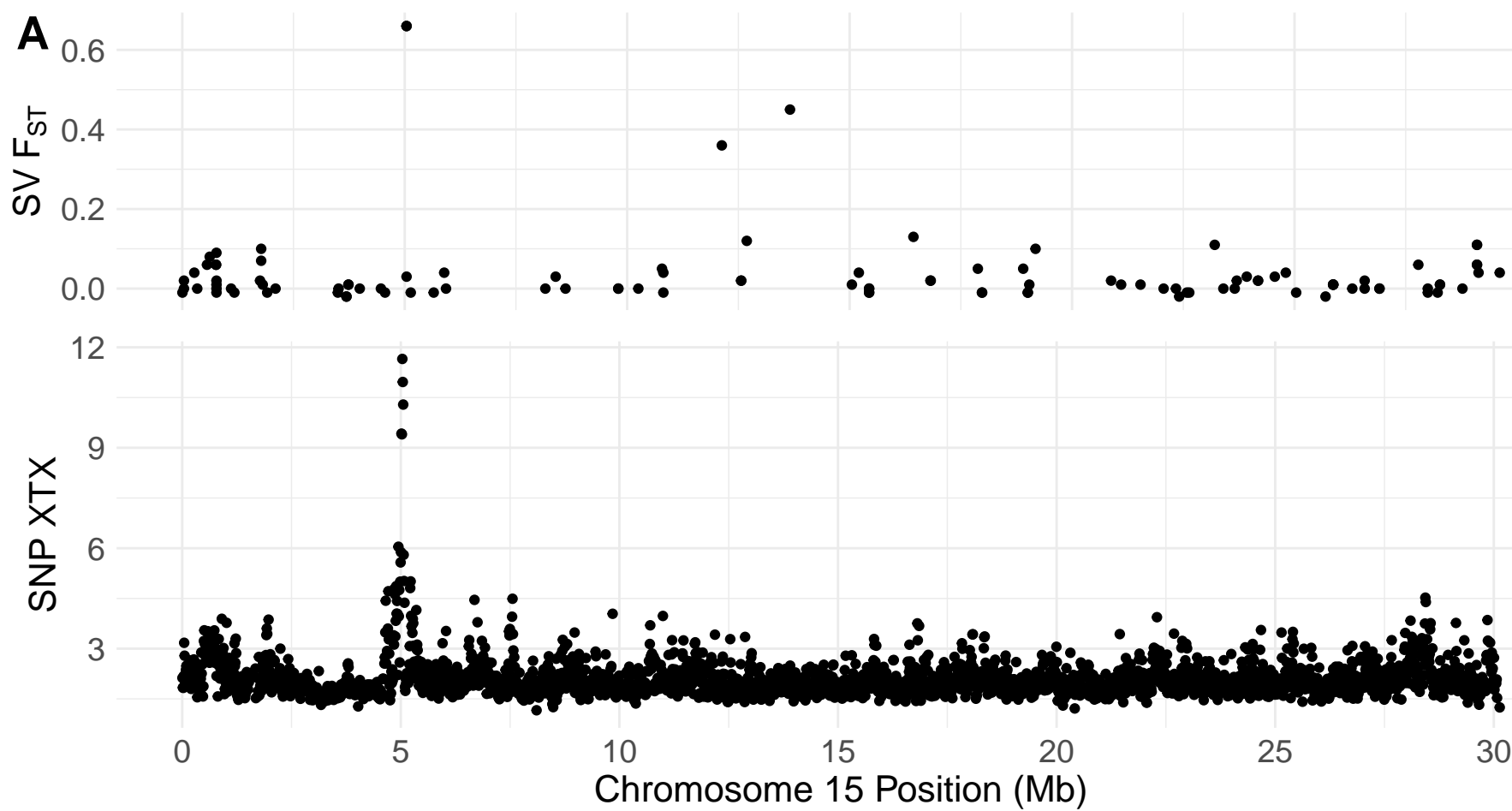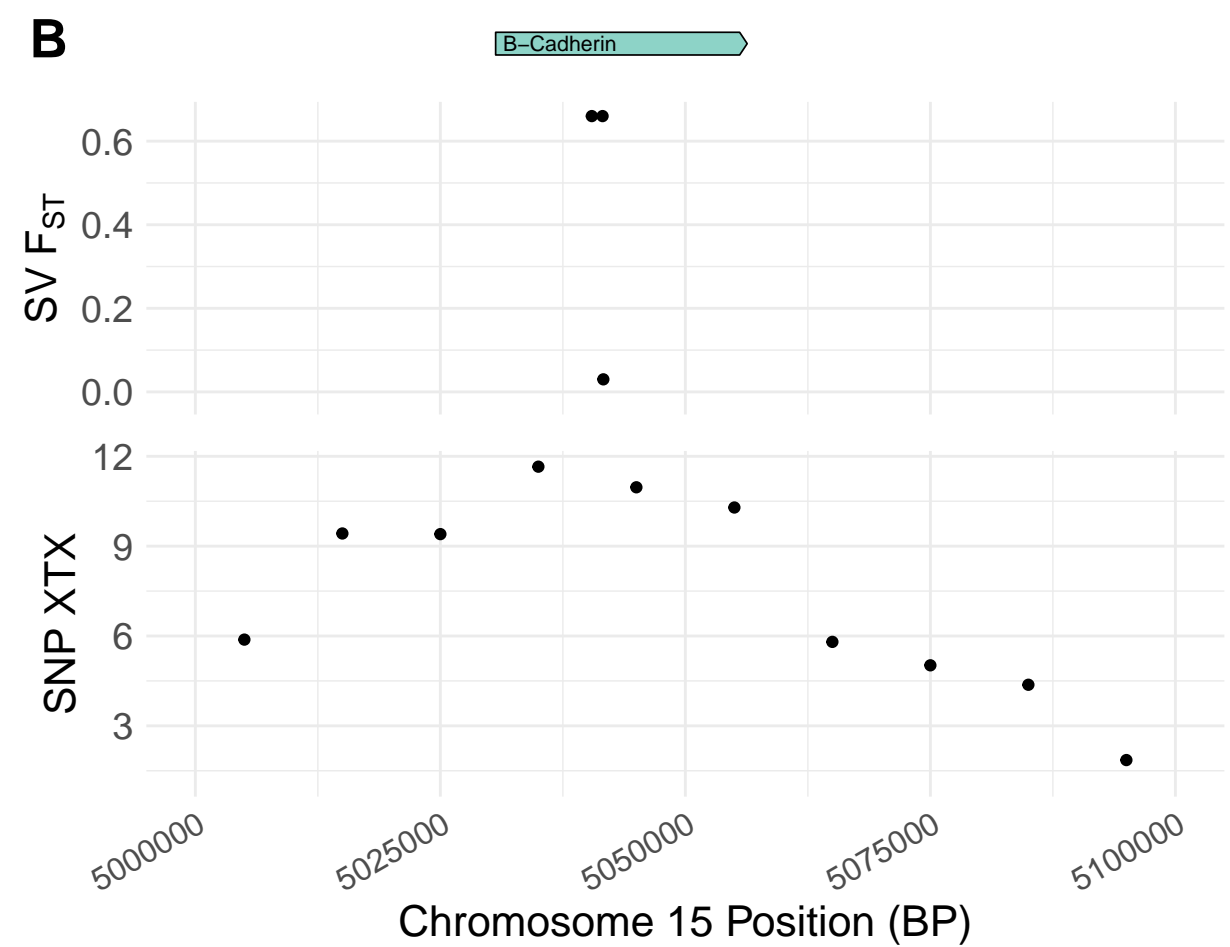

Supplement: S13 Fig — SVs were called between HP and LP populations, highlighting concordance between SV and SNP peaks at ~5 Mb (A). These peaks corresponded with the B-cadherin gene (B) in this region. The SV points at this peak correspond with the breakpoints of a 1,097 bp deletion detected using the software smoove. (PDF) [file pgen.1009566.s013.pdf]

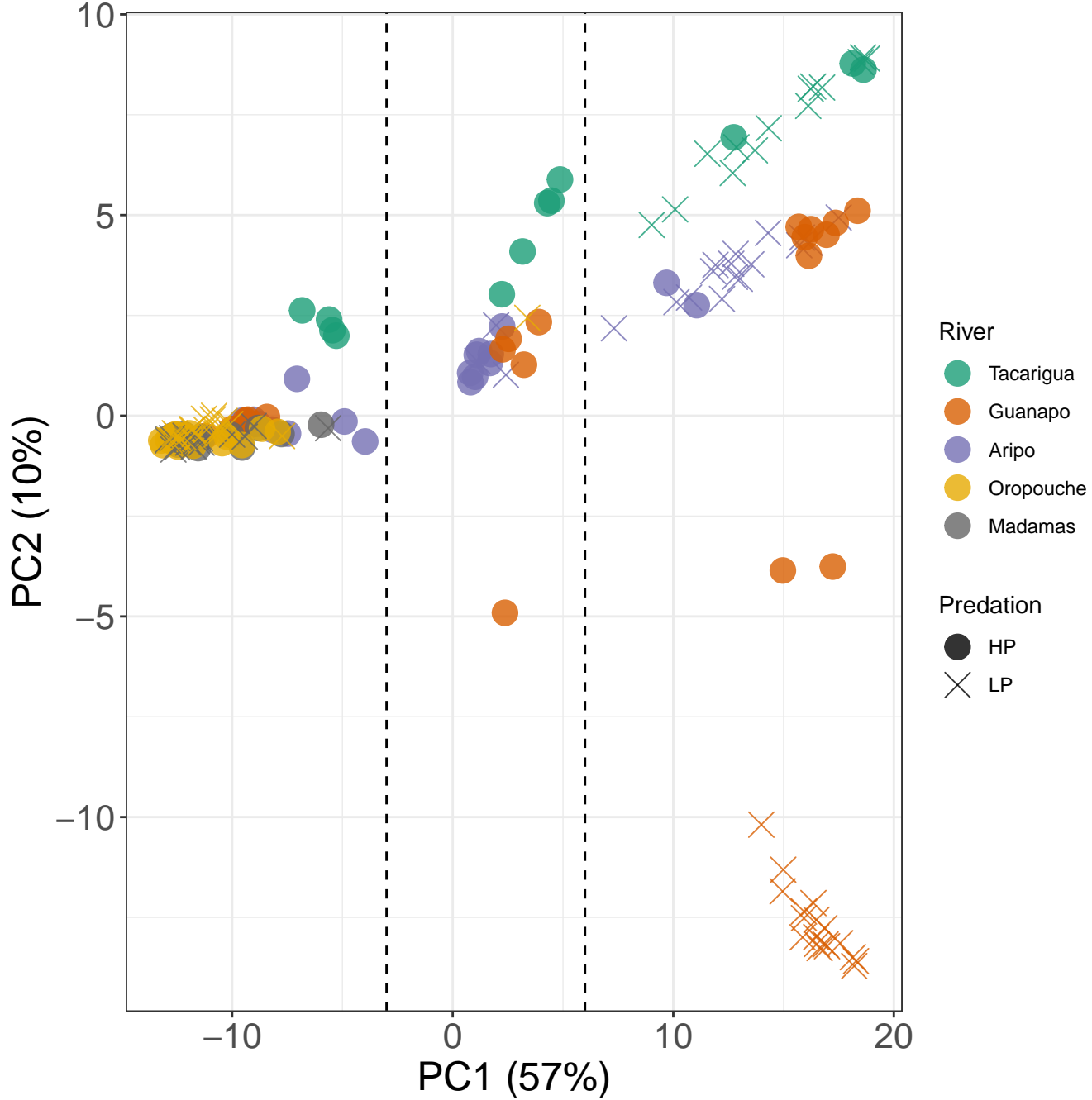

Supplement: S14 Fig — PCA highlights three clusters corresponding to homozygotes (REF and CL), and heterozygotes. Dashed lines denote cut-offs used to define haplogroups. Point colour represents river, and shape represents predation. (PDF) [file pgen.1009566.s014.pdf]

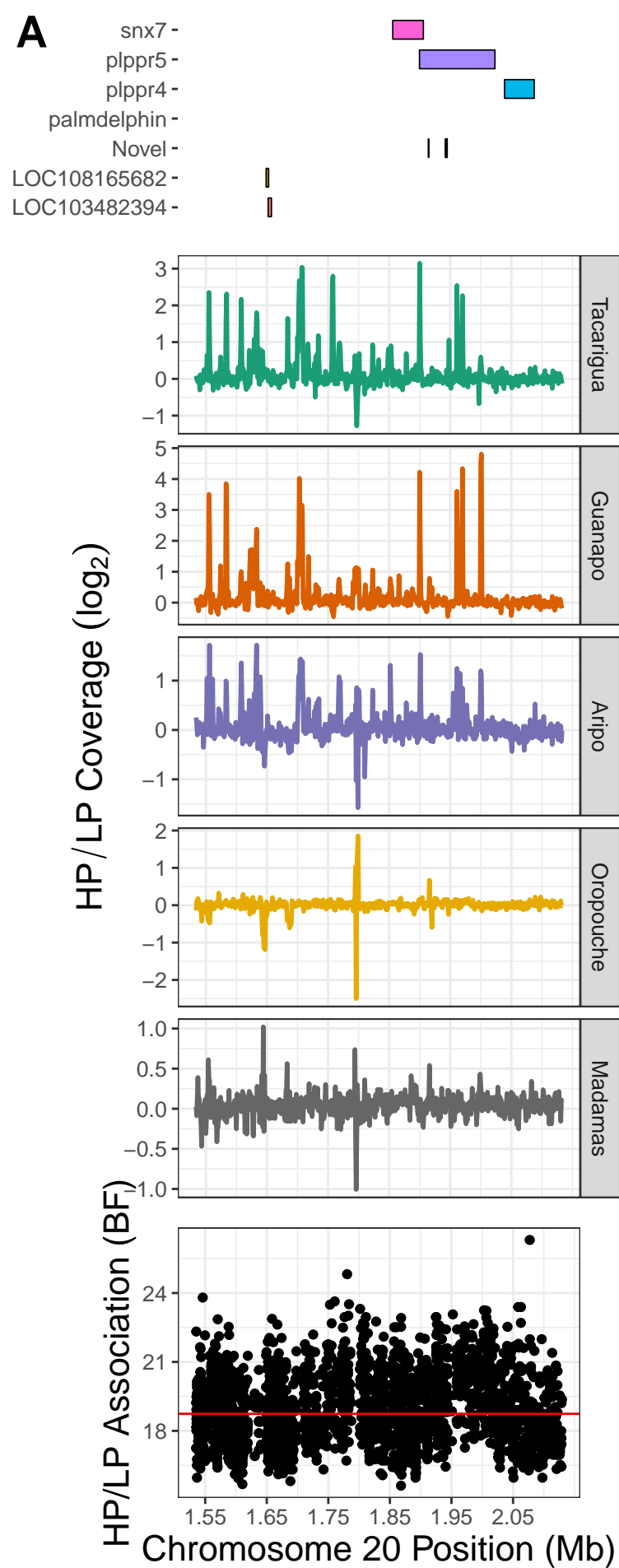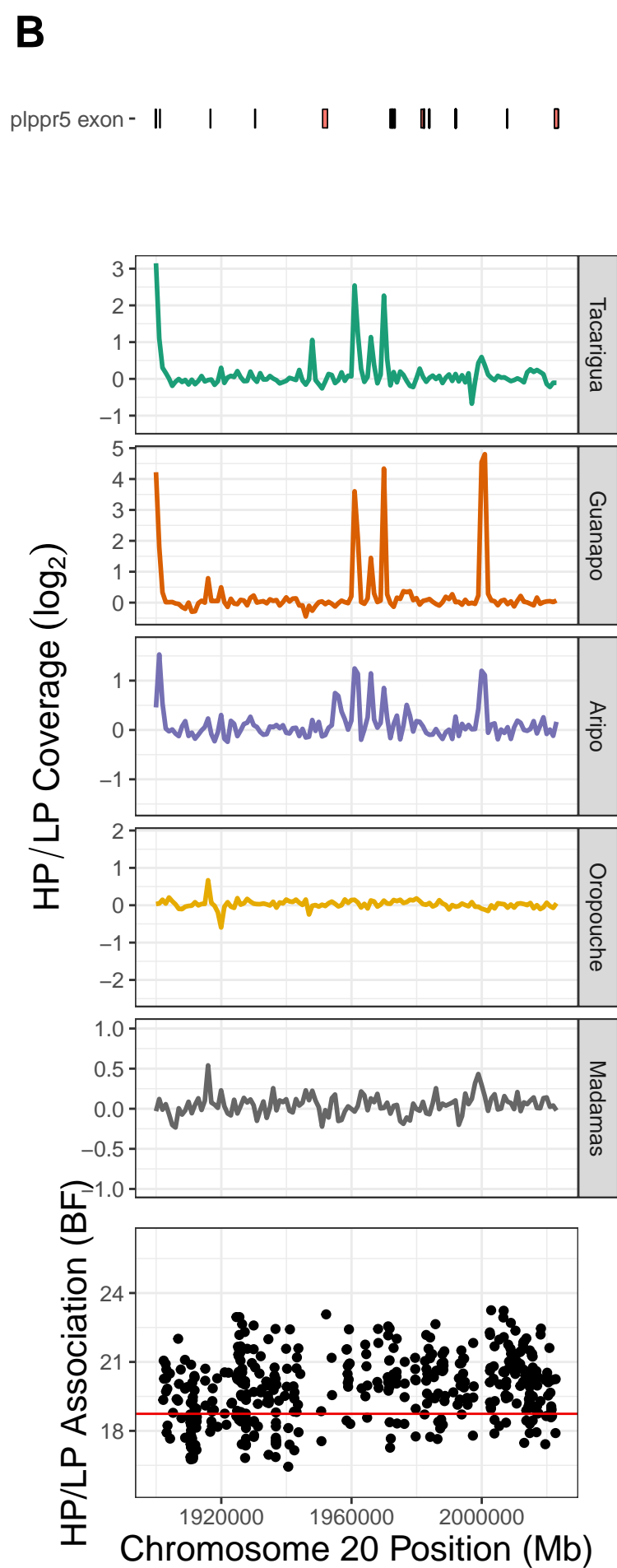

Supplement: S15 Fig — Summary of CL-AP region (A) and plppr5 gene region (B) according to differences in coverage between HP/LP populations and HP/LP association scores per SNP (BF). Of particular note are a peak in HP/LP coverage ratio in Tacarigua, Guanapo and Aripo at ~1.9 Mb (overlapping the plppr5 gene), and the SNP with the highest genome-wide HP/LP association score at ~2.06 Mb (overlapping the plppr4 gene). The peak in HP/LP coverage overlapped with the last exon of plppr5, was driven by reduced coverage in LP populations, and was thus confirmed as a ~1kb deletion in the CL haplotype in all Caroni LP populations by visualising bams in igv. (PDF) [file pgen.1009566.s015.pdf]
