# Supplementary material for: Drainage-structuring of ancestral variation and a common functional pathway shape limited genomic convergence in natural high- and low-predation guppies
Source: PLoS Genet. 2021 May 24;17(5):e1009566. doi: 10.1371/journal.pgen.1009566 (PMC8177651; doi:10.1371/journal.pgen.1009566)
Supplement: S10 Fig — Chromosome 20 co-ordinates are displayed along the upper axis, and scaffold 000094F_0 co-ordinates along the vertical axis. Scaffold 000094F_0 (total length = 1797025 bp) was reversed and inserted at position 836423 on chromosome 20. The proceeding scaffold on chromosome 20 (836423–3164071) was also inverted to reflect higher contact between the start of 000094F_0 and the chr20 region around 3164071. (PDF) [file pgen.1009566.s010.pdf]

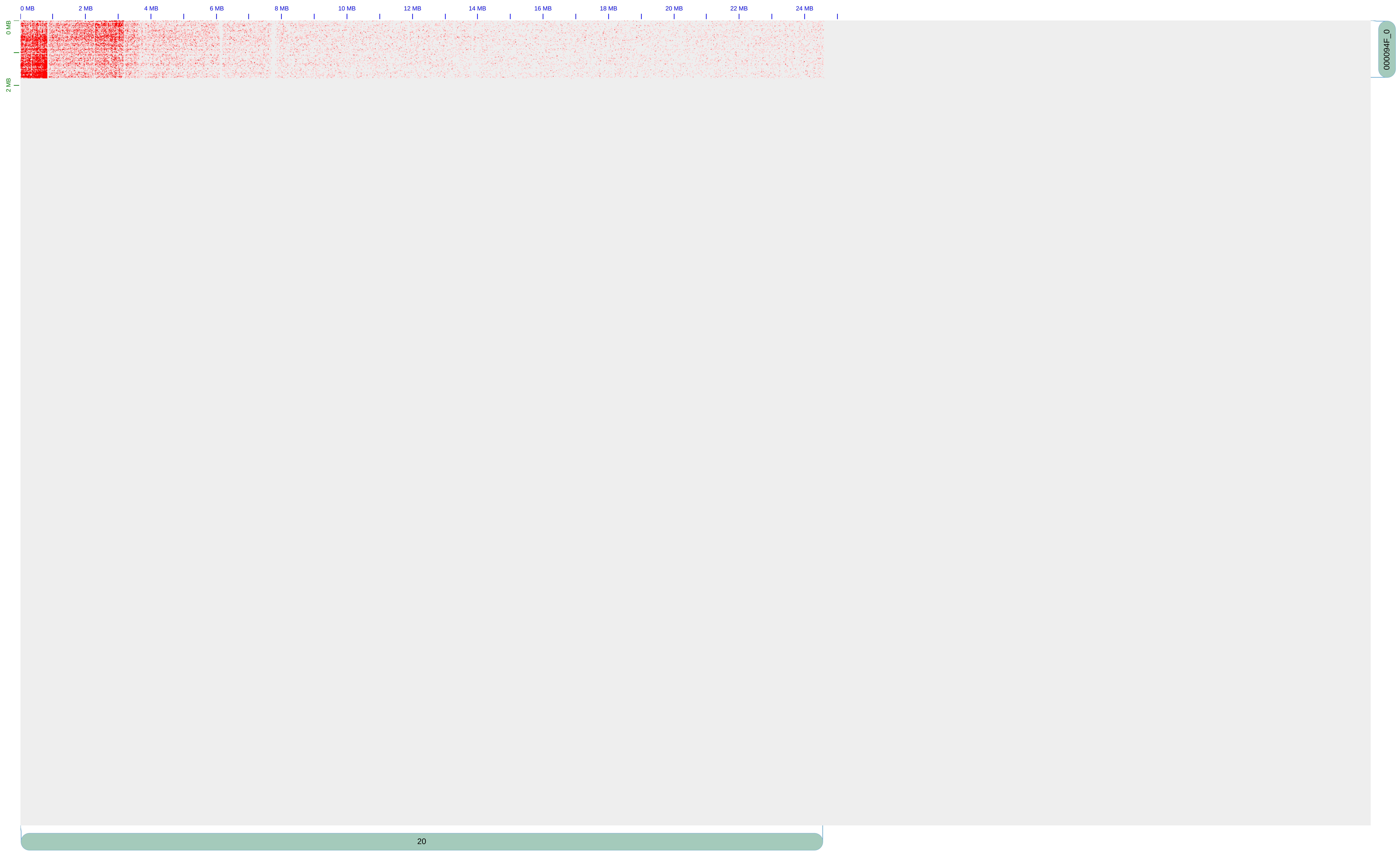

0 MB
2 MB
4 MB
6 MB
8 MB
10 MB
12 MB
14 MB
16 MB
18 MB
20 MB
22 MB
24 MB
0 MB
2 MB
000094F_0
20
