# Supplementary material for: Drainage-structuring of ancestral variation and a common functional pathway shape limited genomic convergence in natural high- and low-predation guppies
Source: PLoS Genet. 2021 May 24;17(5):e1009566. doi: 10.1371/journal.pgen.1009566 (PMC8177651; doi:10.1371/journal.pgen.1009566)
Supplement: Attachment — Submitted filename: plos_genetics_response_to_reviewers.docx [file pgen.1009566.s024.docx]

Drainage-structuring of ancestral variation and a common functional pathway shape limited genomic convergence in natural high- and low-predation guppies

**Response to reviewers:**

The manuscript has been updated to include an Author Summary section, as per PLOS Genetics guidelines. Otherwise, the manuscript is the same as that originally submitted.
