# Supplementary material for: Drainage-structuring of ancestral variation and a common functional pathway shape limited genomic convergence in natural high- and low-predation guppies
Source: PLoS Genet. 2021 May 24;17(5):e1009566. doi: 10.1371/journal.pgen.1009566 (PMC8177651; doi:10.1371/journal.pgen.1009566)
Supplement: Attachment — Submitted filename: guppy_convergence_ms_PlosGenetics_response_to_reviewers.docx [file pgen.1009566.s025.docx]

Dear Dr Whiting,

Thank you very much for submitting your Research Article entitled 'Drainage-structuring of ancestral variation and a common functional pathway shape limited genomic convergence in natural high- and low-predation guppies' to PLOS Genetics.

The manuscript was fully evaluated at the editorial level and by three independent peer reviewers. The reviewers appreciated the attention to an important problem, but raised some substantial concerns about the current manuscript. Based on the reviews, we will not be able to accept this version of the manuscript, but we would be willing to review a much-revised version. We cannot, of course, promise publication at that time.

All reviewers agree that this can be a really interesting contribution to PLOS Genetics but there is some work to do both on clarity of presentation and display items, background information (when available) on the study populations, and specific ideas to further analysis. All of these requests from the reviewers appear very reasonable and should be carefully addressed before resubmission.

Should you decide to revise the manuscript for further consideration here, your revisions should address the specific points made by each reviewer. We will also require a detailed list of your responses to the review comments and a description of the changes you have made in the manuscript.

If you decide to revise the manuscript for further consideration at PLOS Genetics, please aim to resubmit within the next 60 days, unless it will take extra time to address the concerns of the reviewers, in which case we would appreciate an expected resubmission date by email to plosgenetics@plos.org.

If present, accompanying reviewer attachments are included with this email; please notify the journal office if any appear to be missing. They will also be available for download from the link below. You can use this link to log into the system when you are ready to submit a revised version, having first consulted our [Submission Checklist](https://eur03.safelinks.protection.outlook.com/?url=http%3A%2F%2Fjournals.plos.org%2Fplosgenetics%2Fs%2Fsubmit-now%23loc-submission-checklist&data=04%7C01%7CJ.Whiting2%40exeter.ac.uk%7Cbf71176471e144b229c708d8b640947f%7C912a5d77fb984eeeaf321334d8f04a53%7C0%7C0%7C637459735781194607%7CUnknown%7CTWFpbGZsb3d8eyJWIjoiMC4wLjAwMDAiLCJQIjoiV2luMzIiLCJBTiI6Ik1haWwiLCJXVCI6Mn0%3D%7C1000&sdata=APAp3wINuMN7iRX6bL7Ssl%2BxqOKNLMYf0AdrrTmDDMs%3D&reserved=0).

To enhance the reproducibility of your results, we recommend that you deposit your laboratory protocols in protocols.io, where a protocol can be assigned its own identifier (DOI) such that it can be cited independently in the future. For instructions see our [guidelines](https://eur03.safelinks.protection.outlook.com/?url=http%3A%2F%2Fjournals.plos.org%2Fplosgenetics%2Fs%2Fsubmission-guidelines%23loc-materials-and-methods&data=04%7C01%7CJ.Whiting2%40exeter.ac.uk%7Cbf71176471e144b229c708d8b640947f%7C912a5d77fb984eeeaf321334d8f04a53%7C0%7C0%7C637459735781204604%7CUnknown%7CTWFpbGZsb3d8eyJWIjoiMC4wLjAwMDAiLCJQIjoiV2luMzIiLCJBTiI6Ik1haWwiLCJXVCI6Mn0%3D%7C1000&sdata=RyoVif9kmPNe1InnVsi8wBSSyfmNfNMubcOVLQeQzeg%3D&reserved=0).

Please be aware that our [data availability policy](https://eur03.safelinks.protection.outlook.com/?url=http%3A%2F%2Fjournals.plos.org%2Fplosgenetics%2Fs%2Fdata-availability&data=04%7C01%7CJ.Whiting2%40exeter.ac.uk%7Cbf71176471e144b229c708d8b640947f%7C912a5d77fb984eeeaf321334d8f04a53%7C0%7C0%7C637459735781204604%7CUnknown%7CTWFpbGZsb3d8eyJWIjoiMC4wLjAwMDAiLCJQIjoiV2luMzIiLCJBTiI6Ik1haWwiLCJXVCI6Mn0%3D%7C1000&sdata=MY8ztuP6HOsN5gtQ1iwtS4HG8fsjoicRR2KW%2FI9Lml0%3D&reserved=0) requires that all numerical data underlying graphs or summary statistics are included with the submission, and you will need to provide this upon resubmission if not already present. In addition, we do not permit the inclusion of phrases such as "data not shown" or "unpublished results" in manuscripts. All points should be backed up by data provided with the submission.

While revising your submission, please upload your figure files to the [Preflight Analysis and Conversion Engine](https://eur03.safelinks.protection.outlook.com/?url=http%3A%2F%2Fpace.apexcovantage.com%2F&data=04%7C01%7CJ.Whiting2%40exeter.ac.uk%7Cbf71176471e144b229c708d8b640947f%7C912a5d77fb984eeeaf321334d8f04a53%7C0%7C0%7C637459735781214597%7CUnknown%7CTWFpbGZsb3d8eyJWIjoiMC4wLjAwMDAiLCJQIjoiV2luMzIiLCJBTiI6Ik1haWwiLCJXVCI6Mn0%3D%7C1000&sdata=wXsJzLJoeBrYs7pj8x2Pyt3IW2s89eMBs0YG4wWLYkU%3D&reserved=0) (PACE) digital diagnostic tool. PACE helps ensure that figures meet PLOS requirements. To use PACE, you must first register as a user. Then, login and navigate to the UPLOAD tab, where you will find detailed instructions on how to use the tool. If you encounter any issues or have any questions when using PACE, please email us at figures@plos.org.

PLOS has incorporated [Similarity Check](https://eur03.safelinks.protection.outlook.com/?url=http%3A%2F%2Fwww.crossref.org%2Fcrosscheck.html&data=04%7C01%7CJ.Whiting2%40exeter.ac.uk%7Cbf71176471e144b229c708d8b640947f%7C912a5d77fb984eeeaf321334d8f04a53%7C0%7C0%7C637459735781214597%7CUnknown%7CTWFpbGZsb3d8eyJWIjoiMC4wLjAwMDAiLCJQIjoiV2luMzIiLCJBTiI6Ik1haWwiLCJXVCI6Mn0%3D%7C1000&sdata=W%2F6mcHUQduMnPXfLcuAKmGHosld2yUG3l9BQrDkIIus%3D&reserved=0), powered by iThenticate, into its journal-wide submission system in order to screen submitted content for originality before publication. Each PLOS journal undertakes screening on a proportion of submitted articles. You will be contacted if needed following the screening process.

To resubmit, use the link below and 'Revise Submission' in the 'Submissions Needing Revision' folder.

We are sorry that we cannot be more positive about your manuscript at this stage. Please do not hesitate to contact us if you have any concerns or questions.

Yours sincerely,

Mikkel H. Schierup

Associate Editor

PLOS Genetics

Bret Payseur

Section Editor: Evolution

PLOS Genetics

Reviewer's Responses to Questions

**Comments to the Authors:**

**Please note here if the review is uploaded as an attachment.**

Reviewer #1: Dear James and colleagues,

I have enjoyed reading and reviewing your manuscript.

My comments are both brief and minor.

As you note in your manuscript you are working with an elegant system for studying evolutionary responses to different ecological pressures, in this case predation risk.

The system is a great choice for investigating convergent genomic evolution which may underlie repeated phenotypic changes found across the different hp and lp populations.

The sampling scheme and sample size are both well suited to addressing this question.

The introduction contains an excellent overview of the different genomic pathways evolution can take to reach convergent phenotypic outcomes.

The progression through the different analyses is intuitive; first resolving population structure and demographic history, before investigating potential targets of repeated selection across pairs of sister HP and LP populations. We have taken a very similar approach ourselves in a study of coastal and pelagic dolphin populations.

The story is somewhat complex, and I found myself having to re-read sections of the text and scroll back and forth, for example to remind myself of the different abbreviations, river names etc. But I don't have any solution for this and I don't feel this is due to any flaw in the writing or presentation. There just is a level of complexity that requires anyone not already familiar with the study site /system to have to read the manuscript closely. I feel you've made good choices in the level of detail included and the strength of the conclusions drawn. I appreciated the teasing out of the SV underlying the Chr20 peak. Overall, there is no clear functional link between genomic variation and phenotypic variation, but that is often the case with non-model study systems (and could definitely be said about my own papers!). I am sure there will be opportunities to test these associations further perhaps in a lab based setting in future studies. What this study has achieved is identifying some very strong candidate regions for investigation with an equally strong understanding of the underlying evolutionary and demographic history.

I think it is a valuable contribution to our understanding of parallel evolution in nature.

Some minor thoughts as I was reading through the manuscript:

Figure 1D the shapes of different predation regime are not always clear due to dense clustering. Could you use open and filled markers to distinguish HP and LP? and does that make it any clearer?

**>>> We appreciate the differences between HP and LP were not particularly clear. We experimented with the suggestions, however the main issue is relatively tight clustering of rivers within the global PCA space. To make this clearer, we now include a column of river-specific comparisons within the shared PCA space, and mark predation with statistical ellipses. These are the same data as plotted in the main Figure 1D panel, but with the axes constrained in each case to highlight and zoom in on individual clusters. With this we think it is much easier to see the clear separation of HP and LP clusters from the main plot.**

Lines 164-181 I'm more familiar with the significance of D-statistics being expressed as Z-scores as an indication of the number of standard deviations (estimated from weighted block jackknifing) from 0 under the assumption of a normal distribution of shared allele counts with P1 and P1. I'm not familiar with how the estimates and probability scores from Dsuite are derived. Are you able to provide a few more details.

**>>> Based on this comment and comments later on, we have repeated the introgression analyses with an emphasis on the Fbranch statistics. As such, the comment here no longer reflects the revised results. We have however included Z-scores calculated from jackknifing regarding our Fbranch results, which are presented in the main text at lines 170-192. Our main introgression results are also re-estimated on the basis of the true sister species (P. wingei, rather than P. picta), as this data recently became available and allowed us to increase our SNP resolution. The main findings of introgression signatures from this re-analysis are comparable to those originally presented, with the exception of we now report less introgression between APHP and OHP over the CL haplotype (see below).**

Lines 366-368. Work by Simon Martin and colleagues has suggested that the D-statistic is not always suitable for inferring specific introgressed regions within the genome. Simon and colleagues have developed alternative approaches, one most recently with Bill Amos using D estimated from multiple samples per population. Do you think Simon's concerns are relevant here? and would one of these alternative methods be worth applying to your data?

**>>> The reviewer is correct here in saying that D-statistics are inappropriate for assessments of regional introgression. The mentioning of the D-statistic at lines 367-369 was a mistake. In the original Figure S12, we plotted an alternative statistic to D, *fdM*, which is described in *Malinsky et al. 2015 - Science*. This is a variation of Simon Martin’s *fd* is better suited to sliding-window assessments of introgression, as described by Simon here** [**https://github.com/simonhmartin/genomics_general**](https://github.com/simonhmartin/genomics_general)**. However, following our repeated analysis of introgression, using P.wingei outgroup rather than P.picta, we no longer find clear evidence of gene flow between APHP and OHP across the CL haplotype. We believe this original signal was partly confounded by issues with merging the more diverged P.picta data with our guppy data, which led to a bias in the sharing of sites between GHP (the same population as the reference genome is derived) and P.picta (where data was missing for P.picta, the reference genome was assumed). This led to an inflation of ABBA sites in the trio of GHP,APHP,OHP (P4=P.picta), which subsequently looked like gene flow between APHP and OHP. This signal was particularly strong over the CL-AP region, as APHP and OHP are fixed for the REF haplotype over this region whereas GHP is polymorphic for the REF and CL haplotype. We have since resolved these issues of inflated ABBA sites between GHP and the outgroup by including the true sister species (P. wingei) and modifying our SNP-calling pipeline to avoid assuming missing outgroup SNPs are reference genome alleles (HOM REF genotypes). We no longer make reference to introgression at this region therefore, rather we expect the haplotype structure observed in Aripo is the result of double-crossover events (line 417).**

Reviewer #2: The authors present an analysis designed to detect genomic convergence in guppies adapted to alternative environments. Rivers in Trinidad have diverse, predator-rich communities in the higher order portions of streams that drain the Northern Range Mountains. The streams often have waterfalls that exclude predators, but not guppies, from the upper reaches of these streams and tributaries. There is an extensive literature that characterizes how guppies have adapted to life without predators. These adaptations include life histories, morphology, swimming performance, male coloration, male courtship behavior and other aspects of behavior. The question is whether or not there is parallel convergence in the evolution of the genome. If so, then this would provide traction for subsequent studies of the genetics of adaptation. The authors make five paired comparisons between guppies from high and low predation environments in five different rivers. Three of these rivers are part of the Caroni river system on the southwest slopes of the mountains, one is from the Oropuche River system on the southeastern slopes and one is from the Madamas River, an independent drainage on the north slopes of the mountains.

The authors scan for portions of the genome that bear evidence of selection with three different types of paired comparisons between high and low predation populations in each of the five rivers and accept the results as positive evidence of selection if at least two of the three methods support that evidence. They find scant evidence for convergence in the form of genetic differences repeated in all five rivers. The one signal, a weak one, is the signature of selection in genes associated with the Cadherin-signaling pathway. The much stronger signal “mapped to a previously unplaced scaffold…at the start of chromosome 20” (lines 278-282). This is the strongest signal in the analysis, but is only evident in the three rivers that were part of the Caroni river system. They present a detailed analysis of the nature of the differences between the HP and LP population in each river, address whether or not it represents an inversion (it appears to not be an inversion, but may instead be a region of restricted crossing over near the centromere) and discuss the genes contained within this region and the ones that carry the strongest signs of selection.

The results are disappointing because of the absence of strong, uniform signals of parallel evolution of the genome, but the analysis is sound. Such convergent genomic evolution has emerged in studies of other classic examples of convergent evolution, most notably the sticklebacks, and the authors discuss why guppies might be different from sticklebacks. This paper includes a thorough analysis of population demography and gene exchange among populations. One feature of guppies that distinguishes them from sticklebacks and offers a potential explanation is that the freshwater populations of sticklebacks are derived from a large, panmictic marine population and appear to have been established by a large number of colonists, so each one begins with a good sampling of the genetic variation from the founder. In guppies, the ancestral populations are genetically distinct from one another and the descendant populations often carry a signature of a genetic bottleneck, meaning that they were established by a small number of founders. Overall, I think that the paper represents a significant step forward in our understanding of the system and merits publication. I do, however, feel that many improvements could be made to the presentation.

**>>> We would like to point out that convergent guppy phenotypes being derived through limited genetic convergence is neither more or less disappointing than strong genetic convergence. We believe our results frame the guppy system as an important model to investigate the limitations of genetic convergence and the ability of adaptation to overcome these.**

General Comments:

1. They must provide better locality data for where the fish were collected from. There are multiple candidates for low predation in all five streams. We need to know which one they chose since they each could have different histories and properties. If nothing else, posterity needs this information if anyone is to follow up on this work. I am especially concerned about the Aripo River since one of the LP tributaries is a site where guppies were deliberately introduced by John Endler in 1976. Their recent origin and having had what was likely an unusually large number of founders would possibly account for the difference between the Aripo and the other two from the Caroni in the results for chromosome 20 (as highlighted on lines 416-418). I might add that this paper could be a good deal more interesting and important if these fish were indeed from the introduction site.

**>>> We have now included locality information in Table S1, along with information on the previous phenotyping work that has been done on these populations. We hope that in lieu of a quantitative analysis of phenotypic convergence among these populations, this information at least highlights that the populations sampled and studied here at the genomic level do indeed differ in expected HP-LP phenotype. The Aripo population sampled was not associated with the Aripo introduction site. This is now made clear at line 116. Because this study is specifically an assessment of natural populations, the Aripo introduction site would be beyond the scope, however genomic analyses of other guppy introductions are underway or have been recently published eg. https://www.biorxiv.org/content/10.1101/2021.02.10.430609v1.**

2. Polarization of the direction of evolution: Lines 411-414 state that HP is ancestral and LP is derived plus speculates on why we do not see repeated patterns of molecular convergence across drainages. I think this is an important point that needs to be amplified. First, do their data enable them to address whether or not HP really is ancestral to LP? The fact that HP has higher genetic diversity is not by itself adequate. If we imagine HP as being the composite of separate LP populations distributed through the diverse headwater streams that combine to form HP, then their mixture provides an alternative explanation for the patterns of genetic variation. While I consider this top-down alternative to be unlikely, I think it would add to this paper if their data can be martialed to resolve the ancestral-descendant relationship among populations within each of the five rivers. I don’t know if it is possible, but it would be a great contribution if there were some way to nail down who was ancestral vs. descendant. I strongly favor their interpretation, but it would be great if their data could provide some definitive support for it.

**>>> We agree that the evidence of reduced diversity in the upstream populations is not, in itself, evidence that the LP populations are descended from HP sources. We would contest whether the question of HP being ancestral to LP is necessarily the right way of framing the question, and whether this is important for the study of convergent evolution. If the question is whether LP individuals were founded from HP populations, or whether HP populations are sinks of genetic variation from many upstream populations, both may be likely. In truth, neither the present-day HP or LP population is the ‘ancestral population’, but rather both are descended from a shared common ancestor. The question can then be reframed as to which of these populations is a closer representation of the common ancestor. Analogously, in comparisons of marine-freshwater stickleback, contemporary marine populations are an assumed representation of the ancestral population as freshwater populations exhibit elevated genetic drift that has taken them further from the common ancestral state. We would therefore argue that a similar case can be made here with our data presented in Figure 1D (PCA of all populations), discussed now at lines 157-161.**

**Here, we assume all sampled individuals coalesce back to a common ancestor at the global centroid, or rather that their current positions within PC space are mostly the result of drift following separation. Taking this perspective, it is clear that all LP populations (with the exception of Madamas, that shows only very weak HP-LP separation) have exhibited greater drift from the global common ancestor than their respective HP population. This is highly suggestive that the LP populations represent derived states, and the HP populations are closer to the ancestral state. The status of LP populations as the more derived populations would also agree with bottlenecks occurring following separation from HP populations, which we observed in demographic modelling.**

**Ultimately, it is not possible to definitively state which population historically founded the other, and to address this question specifically it would be necessary to sample all upstream sites that drain into the HP population which is beyond the scope of the current study. However we hope that the arguments presented (now also discussed in the text at lines 482-488) provide a more compelling case for the HP = ancestral, LP = derived statuses. We also appreciate that is an important element of our model to explain reduced genetic convergence and believe the improved rationale presented makes for a stronger case.**

3. Clarity of the figures: I am a genomics novice and I found some of the figures to be uninterpretable. I found better trained colleagues who were able to figure them out for me, but if this paper is to be accessible to a general audience then some additional explanation is required.

Fig. 1e: In my discussions with colleagues it emerged that these figures may have been over-interpreted in the text. The apparent gene flow between Madamas, Oropuche and Aripo may not mean that there has been movement from both Madamas and Oropuche into Aripo. It is also possible that there is movement from Madamas to Oropuche then from Oropuche to Aripo. If this is the case, then you should clarify the extent to which these results lend themselves to alternative interpretations. Elsewhere you say that there is evidence of gene flow between Aripo LP and Oropuche LP, but I see no evidence of that in this figure.

**>>> We appreciate that we could have been clearer regarding the interpretation of our introgression analyses. The reviewer is correct in saying that significant D-statistics do not imply introgression has taken place between those populations, but rather introgression has taken place at some branch within the clade and is detected in all downstream positions of the clade. To resolve this and make our results clearer, we now include an additional fbranch analysis that is better able to estimate at what branch within the clade the introgression event took place. The fbranch statistic is described in the original Dsuite paper (see - *Malinsky et al 2020, Mol Ecol Resources*). The results of this are described in lines 170-192 and in Figure 2, and provide clearer inference as to the specific populations involved in introgression within the tree. The reference to introgression between APLP and OLP was in reference to gene flow between APHP and OHP/OLP, and has been amended to reflect this.**

Fig. 1f: I need a more detailed explanation of what the entry in each cell is and a key to what the color coding means, plus an explanation for those cells that are gray and presumably have no entry. My colleagues were able to explain this figure to me but I had no prayer of figuring it out on my own.

**>>> We have updated the figure legend to include additional information regarding Figure 1F, and have included fill legends within each figure.**

Fig. 2a: I again could not understand Fig. 2a on my own but my colleagues were able to explain it. The caption is not adequate for someone without a better genomics background than mine.

**>>> We have updated the figure legend with additional information on the upset plot.**

Fig. 2c and d: You show some compelling results for chromosome 8 but I saw no more about this in the text. What happened here?

**>>> We explored chromosome 8 further in a similar context to chromosome 20, however the evidence for selection in multiple rivers was generally weaker. The reason we visualised this chromosome in Figure 2D was to highlight the relative strength of signal at chromosome 20 compared with the second strongest signal at chromosome 8. Chromosome 8 has the second largest cluster of BayPass windows (7 windows), after chromosome 20 (54 windows), but visualising the pattern within each chromosome makes clear that the signal at chromosome 20 is far stronger and wider (which is not immediately clear in Figure 2C, apart from having a taller peak). There is information regarding the chromosome 8 region and genes within it in Table S6-7. These tables also makes clear that there is some evidence for selection in 4 of the 5 rivers across the chromosome 20 region, whereas the chromosome 8 signal only exhibits selection evidence in Guanapo and Aripo. We felt on the basis of relative signal compared with chromosome 20 it did not make sense to give this region an equivalently detailed analysis. We do however appreciate that this rationale could have been better put across in the text, and it is now included at lines 287-300.**

Fig. 3: I had no problem with this one. Fig. 3b seems to report some differentiation between LP and HP in the Oropuche River in this same portion of chromosome 20. What is going on there?

**>>> Figure 3B shows the results from localPCA analysis performed within each population. This analysis therefore is not making comparisons between HP and LP, but rather is visualising that the region exhibits abnormal population structure relative to that observed across the rest of the chromosome. This is usually due to inversions, reduced recombination, or gene density (see *Li and Ralph 2019 - Genetics*). The purpose then of showing all of these panels is to highlight that, despite the CL haplotype being absent outside of the Caroni drainage, both Madamas and Oropouche also exhibit comparable localised population structure signals at the same region. If the CL haplotype and the REF haplotype represented a unique inversion, we would only expect to observe these signatures in populations with the CL haplotype. That we don’t see this suggests (but does not confirm) that something besides an inversion, like reduced recombination close to the telomere (guppy chromosomes are acrocentric), is a viable alternative to explain the maintenance of these diverged haplotypes in the absence of an inversion.**

4. Discussion: The discussion is not optimally organized. It is a collection of paragraphs on different topics, all appropriate for a discussion, but all presented in a continuous fashion that is hard to follow. I think it would benefit from being divided up into subheadings:

**>>> We thank the reviewer for their constructive feedback here, and have adopted the suggested structure below.**

Paragraph 1 (lines 401-418): an appropriate summary

Paragraphs 2 – 4 (lines 420-477) detail the results for Chromosome 20 and fit well into a single subheading. Some of the paragraphs are overlong and group material that would be better divided into more and simpler paragraphs.

Paragraphs 5-7 (lines 479 – 510) detail population structure, introgression and how structure might explain the scarcity of genomic convergence. You return to this theme in Paragraph 9 on lines 534-542 so perhaps grouping it with these paragraphs under one subheading would make more sense.

Paragraph 8 (lines 512-532) deals with the Cadherin pathway and the different paths to genetic convergence so it seems better to group it under its own subheading.

Additional Comments:

6. Lines 203 – 207 and Fig. S1: The three Caroni River tributaries all have the potential for facilitated gene flow because they are connected and this is reflected in Fig. 1, so why do the steeper mountains that separate these tributaries matter?

**>>> The steeper mountains are important in preventing direct gene flow between upstream LP populations during flooding events. This is a phenomenon that is observed in the Northern drainage, for example between the Paria and Marianne rivers (see *Blondel et al. 2019 - Ecology & Evolution*). The reviewer is correct in suggesting that, physically, Caroni rivers are connected, however gene flow between LP individuals moving downstream and then back upstream through HP sites is unlikely, particularly given how biased migration is downstream generally. This is also reflected in the lack of strong introgression between LP populations in the Caroni drainage. However it cannot be ruled out, so we make note of it at lines 213-214.**

7. Lines 214-216: They are no doubt correct in concluding that there is more evidence of gene flow from LP to HP sites within each river, but they also assume, as have others, that HP is ancestral to LP, meaning that migrants from HP founded the LP populations. This is where it would be great if your data could in some way address who is ancestral and who is descendent within each stream.

**>>> See comment above, this cannot be resolved definitively but all available evidence agrees with our interpretation.**

8. Lines 334-6: I think this statement will be a mystery to anyone who is not very familiar with the guppy literature. It is worth emphasizing that the fish from the Oropuche river system are sufficiently different from those in the Caroni and north slope to have been named a different species by one investigator. But isn’t this pattern consistent with the CL haplotype having arisen within the common ancestor of the Caroni river populations then moved into the Oropuche via gene flow?

**>>> We have endeavored to make reference to the differences among drainages throughout the manuscript, and the phylogeny is presented in Figure 1. Due to additional analysis to resolve the history and dates of separation (Figures 1B), we now mention the P. obscura claims at lines 496-499. Further, based on comments from reviewer 3, we have attempted to better resolve the evolutionary history at the CL region, and present this as FIgure 3D-F. The lack of gene flow that we observe between rivers, in particular out of the Caroni drainage, makes it unlikely that the REF haplotype has been introgressed into the Oropouche, particularly as it would then have had to move from the Oropouche into the Northern drainage (the evidence we have is strongly suggestive the direction of gene flow between Madamas and Oropouche is Madamas > Oropouche, see new Figure S4). The opposite may be possible, i.e. the REF haplotype represents the ancestral Northern/Oropouche haplotype, and it has been introgressed into Caroni where it has spread among HP sites. However this also seems unlikely given how limited gene flow appears to be between Caroni HP sites. The other option, which we suggest at line 463, is that there is strong selection acting on the haplotype that may have limited divergence between the REF haplotypes found in Caroni and Madamas/Oropouche. In our opinion, this is the most likely scenario as it allows for all haplotypes to be ancestral, whereas other explanations require gene flow to occur across multiple rivers which we believe unlikely.**

9. Lines 391-392: Is “lost” the correct word? Might “absence” be better since it implies that the CL haplotype could also have originated in the common ancestor of the Caroni River populations? I either have missed something in your explanation of these results or you are not considering this alternative explanation.

**>>> In regards to this hypothesis, we have attempted to assess the divergence of the CL haplotype in relative terms. These results are described at lines 353-362. In short, the evidence we have is that the CL haplotype is at least as old, and probably older, than the separation between Caroni and Non-Caroni drainages. This makes it unlikely that the haplotype arose after the separation event, else we would expect the divergence between the haplotypes to be less than that of the chromosome-wide signature between APLP and MADHP/OHP (which share an ancestral node). We have however noted the alternative explanation at lines 461-469.**

Reviewer #3: Review for: “Drainage-structuring of ancestral variation and a common functional pathway shape limited genomic convergence in natural high- and low-predation guppies”

General comments:

Instances of convergent and parallel evolution facilitate the discovery of common rules of organismal diversification. In this study, Whiting et al. explore genetic basis underlying convergent evolution of Trinidadian guppies adapted to high- and low- predation (HP and LP) pressures. The authors sampled ~15 HP and ~15 LP individuals from five rivers and obtained whole genome data at ~10x coverage. Overall, I think the core questions are interesting. The study system, the sampling scheme, and the genomic dataset are well suited to address the questions. In terms of genetic convergence, the authors find a haplotype on chr20 and genes involved in cadherin signalling appear to be involved to some degree, but not much else, and nothing at the level of individual genes or variants. The authors conclude that this is because of a lack of shared genetic variation between LP populations. The writing is generally clear and possible to follow, but not always – it would benefit from some improvements – on occasion, the expressions and terminology used sound somewhat clumsy, and there is too much repetitiveness. I have two major concerns and a number of comments and suggestions for the authors to consider before publication.

Major comments/concerns:

First, I find that the paper lacks background information. This limits my ability to understand if the methods employed are appropriate and also how the paper fits into the broader context of other empirical studies of convergent evolution.

Second, the conclusion that the paucity of genetic convergence is because of a lack of shared genetic variation is speculative. The authors did not test this in any way; all they have is indirect evidence in the form of inferred bottlenecks.

**>>> We have replied to the specific comments below in respect to these two comments.**

I am going to expand on both of these points with specific comments/suggestions .

Specific comments/suggestions:

In the introduction, and in fact anywhere in this paper, I am missing information about the timeframe of evolution of these convergent phenotypes. Is anything known about when the waterfalls separating the HP and LP populations formed? At least a ballpark figure – 50 years, 5000 years, 5 million? And how much does this differ between the rivers? The ages of the rivers? Or some inference from the genetic data?

**>>> We have now included rough estimations as to the separations within and between rivers in Table S2 and at lines 139, 150,1 59. We have attempted to date the phylogenetic splits using an implementation of SNAPP that is time-calibrated based on the divergence with the sister species P. wingei (see methods line 634-655). This is a good approach for estimating the phylogeny of the meta-population, as the species tree is inferred by integrating over all possible gene trees rather than separately inferring gene trees for all markers. A benefit of this then is being able to build a well-supported consensus despite the expectation that some gene trees differ from the species tree due to gene flow or incomplete lineage sorting. However we urge caution regarding these estimates; they provide useful ballpark figures, at least on the scale of orders of magnitude, however variation among divergence estimates among rivers are likely to still be influenced by contemporary gene flow as much as as actual differences in age of splits. These considerations are described at lines 488-493. We have also included a brief discussion of how our results compare with previously published estimates (lines 488-503), which we believe provide important and useful context for researchers working on the guppy system.**

Another thing that is missing is some idea as to how far has the adaptation proceeded in the five rivers. If the relative ages of the waterfalls are different, perhaps the LP populations are adapted to different degrees? Also, even if the ages are the same, are the phenotypes always the same? Have these specific populations been phenotyped previously? Could some phenotyping (e.g. colour?) be done for the current study?

**>>> The populations have been extensively phenotyped both in wild-caught individuals and lab-reared individuals. Citations for these studies are now included alongside sampling localities in Table S1. We have also updated the text with recent information on colour phenotypes at lines 104-105, which are generally complex. Overall, a comprehensive comparison of convergent phenotypes is out of reach for this paper and has been well-documented elsewhere.**

**Given the estimates we have for separations between HP and LP populations are on the order of thousands of years (see above), we do not believe it relevant to discuss whether LP populations are adapted to different degrees. Given that evolution of LP phenotypes are observed after a handful of generations in experimental populations (see refs at line 111), the ages we observe for HP-LP splits here are expected to be more than sufficient for adaptation to have taken place. Further, the system is complicated by different rates of gene flow between HP and LP populations that is as likely to influence rates of adaptation as the relative ages of populations. It cannot be argued therefore that older populations may be ‘more’ adapted, given ongoing gene flow.**

Lines 135-142: “PC2 (19.8%) separated out Caroni rivers, highlighting population structure within this drainage is stronger than structure between Madamas and Oropouche, despite these rivers being in separate drainages…….”

- This may also be simply a sample size effect – I suggest you try to verify this by doing a PCA including only two Caroni rivers and Madamas and Oropouche.The same for fineSTRUCTURE. I suspect that your conclusions may change.

**>>> We have repeated the PCAs keeping only 2 (all 3 variations) of the Caroni rivers, and find that our conclusions remain the same (Figure S2). This is in keeping with the rest of our analyses, in particular those that highlight both increased migration within the Madamas and Oropouche rivers, and also the strong signature of introgression between them. The latter provides a mechanism to explain why we would expect signatures of population structure to be weaker between the Madamas and Oropouche rivers. The lack of any strong gene flow among the three Caroni rivers also provides a mechanism that explains why these rivers might be expected to be more strongly structured than the Madamas and Oropouche rivers. We do not think it is necessary to similarly repeat the finestructure analysis given the robustness of the PCA results to the removal of individual Caroni populations and the weight of various lines of evidence for this result.**

Lines 164-181 and Figure 1E:

- I suggest that you plot and cite in text the f4-ratio statistics, not the D. The f4-ratio estimates the admixture proportion, and thus, unlike D, it is comparable between studies in a biologically meaningful way as a measure of gene-flow strength. On the other hand, the magnitude of D depends on the amount of incomplete lineage sorting.

**>>> We now report the f4-statistics alongside the D results in the main text. In response to this comment, and those of reviewer 1, we have included an Fbranch analysis in order to estimate introgression involving specific populations (figure 2). This now replaces Figure 1E. The Fbranch is derived from the f4-ratio (the f4-ratio is partitioned into nodes along the phylogeny, so this statistic is similarly biologically comparable across systems as suggested by the reviewer.**

Figure 1B,C, and F –

All three panels lack scale bars. For 1B, perhaps a proper phylogeny would be more appropriate than the fineSTRUCTURE clustering? And for 1F, I wonder why the authors did not use the unfolded site frequency spectrum, which would be more informative and perform better in fastsimCoal2. The authors did add an outgroup to the VCF for the Dsuite analyses, so they could use it to polarise ancestral-derived alleles.

**>>> We have now included the scale bars for Figure 1C and 1F (now 1E). In terms of the phylogeny, the goals of this were to determine whether HP-LP populations are sister groups, and so independently derived, and highlight relationships among the rivers. Whilst we believe that goal was achieved partially by the finestructure and PCA analyses, we have now included a dated SNAPP phylogeny which addresses this comment and the earlier comment regarding age of splits. We used the folded SFS because the unfolded requires accurate assessment of the derived allele frequency, which in turn requires data from two outgroups (https://pubmed.ncbi.nlm.nih.gov/29769282/). The sensitivity of the unfolded SFS to bias (as discussed recently in Momigliano et al. 2021) was the major driver behind choosing the folded SFS.**

Maybe most importantly, given the conclusion that of a lack of genetic convergence is due to a lack of shared genetic variation, it would be interesting to know how much shared genetic variation there is among the five LP populations. Maybe create a 2d site frequency spectrum for pairs of LP populations?

**>>> We thank the reviewer for this constructive suggestion and have now included the recommended matrix of 2D SFS among LP populations. This approach indeed confirms limitations on shared variation among LP populations (particularly TACLP and GLP), with the exception of MADLP and OLP due to introgression.**

The selection tests raise two thoughts:

a) The methods – both XtX and AFD reflect allele frequency shifts in slightly different ways. XP-EHH is different, reflecting recent selection. Since the authors state “Putatively selected windows were identified if they were detected as outliers by at least two approaches” – I wonder how often this simply reflects an agreement between the two allele-frequency-based approaches?

**>>> This information is now available in Table S4. Here we describe the total number of outliers for each singular scan, and then the numbers of outliers that overlapped in any pair or all 3. What this shows is that our analysis isn’t biased towards AFD/XTX outliers alone, but rather most outliers are either AFD/XTX or AFD/XPEHH. This is to be expected, given the strongest signals of XTX (selective sweeps for alternate variants) are not expected to produce differences in extended haplotype homozygosity, as both populations exhibit sweep-like signatures of increased homozygosity). However both of these processes involve relatively large changes in allele frequencies, thus our analysis can be considered to be largely confirming XTX and XPEHH scans on the basis of reasonably high AFD change. We do however also observe numbers of regions that overlap in all three scans, and regions that overlap only in XTX and XPEHH. In general however, there is no evidence that our selection scan outliers are biased to only AFD/XTX outliers only as suggested. This is an important consideration, and we thank the reviewer for their suggestion.**

b) With fixed 10kb windows, how much are the results driven by stochasticity in the number of SNPs per window? The windows with fewer SNPs will have greater variance and present outliers more often. Perhaps trying to apply the analysis with windows that have equal numbers of SNPs would be interesting?

**>>> Based on this comment we clarified what the effect of SNP density on our results might be. Specifically, we looked at whether our overlapping outlier windows, from which we derive conclusions regarding the cadherin-signalling pathway enrichment, and our baypass outlier windows exhibit less SNPs than randomly drawn groups of windows of the same count number. For example, we have 153 10kb selection scan outlier windows that are overlapping among rivers, so we repeatedly (N=10,000) randomly sampled 153 windows from our total set of genome-wide windows that included at least 1 SNP and compared the permuted distribution of median SNP counts to our observed. In each case (Figure S6), we find that our windows of interest tend to have more SNPs on average than equally sized groups of randomly drawn windows (one-tailed test, permuted p = 0.0172 and 0.0176). The opposite to what was suggested. Based on this, we do not believe our overlapping outlier results or baypass results are influenced by variance associated with lower SNP counts. Rather, our windows of interest tend to have more SNPs than the genome-wide average, indicative of good quality of data within these regions. We have made a note of these results in the text at lines 244-245 and 272-273, and include the results in Figure S6.**

I liked the permutation tests for pathway enrichment.

Language, typos etc.:

Lines 44-45: “convergent phenotypes are often underwritten by convergent changes at the genetic level”

Underwritten sounds a bit like we are reading about insurance or finance. How about “convergent phenotypes are often encoded by …. “, or something like that?

**>>> Corrected**

Lines 69-70: “Given the range of genetic convergence observed across empirical studies, the importance of different contingencies have emerged.”

- How about: “Given the divergence in modes and levels of genetic convergence observed across empirical studies, different contingent factors have emerged as important.”

**>>> We have incorporated this suggestion, but have opted to replace “divergence” with “differences”, because of potential confusions surrounding divergence as the opposite of convergence (line 69).**

Line 72-73: “Limitations in this map are expected to upwardly bias reuse of the same genes or mutations, but redundancy can allow for the evolution of different genes in shared functional pathways.”

- This takes some deciphering. Why “limitations in this map”? Do you mean “limitations in the redundancy of this map”? Why “bias”? Why “allow for”?

- Maybe you mean something like this: “Simple, e.g. one-to-one, mapping is expected to result in reuse of the same genes or even mutations, while redundancy can lead to convergent phenotypes for example by selection on different genes in shared functional pathways.”

**>>> We have incorporated this, slightly edited, into the text (line 72-75).**

Lines 80-83: “whereby consistencies in the multidimensional fitness landscape channel adaptation along conserved paths. These latter two limiting factors may also explain why genetic convergence can vary for the same traits in the same species in global comparison.”

- I don’t understand any of the logic here – if consistencies in fitness landscape channel adaptation, why does genetic convergence vary?

**>>> We have attempted to make this clearer in the main text. The original meaning was that if consistent channels adaptation along conserved paths, inconsistencies may prevent this.**

Many similar cases follow, but in general the paper is possible to follow.

Much of the discussion repeats the results. I suggest cutting down the repetition…

**Have all data underlying the figures and results presented in the manuscript been provided?**

Large-scale datasets should be made available via a public repository as described in the *PLOS Genetics* [data availability policy](https://eur03.safelinks.protection.outlook.com/?url=http%3A%2F%2Fjournals.plos.org%2Fplosgenetics%2Fs%2Fdata-availability&data=04%7C01%7CJ.Whiting2%40exeter.ac.uk%7Cbf71176471e144b229c708d8b640947f%7C912a5d77fb984eeeaf321334d8f04a53%7C0%7C0%7C637459735781224594%7CUnknown%7CTWFpbGZsb3d8eyJWIjoiMC4wLjAwMDAiLCJQIjoiV2luMzIiLCJBTiI6Ik1haWwiLCJXVCI6Mn0%3D%7C1000&sdata=jj7FRNtJsfAOicNcMwGBbEZya4OdGQz38mBqSL%2FawrU%3D&reserved=0), and numerical data that underlies graphs or summary statistics should be provided in spreadsheet form as supporting information.

Reviewer #1: None

Reviewer #2: Yes

Reviewer #3: None

PLOS authors have the option to publish the peer review history of their article ([what does this mean?](https://eur03.safelinks.protection.outlook.com/?url=https%3A%2F%2Fjournals.plos.org%2Fplosgenetics%2Fs%2Feditorial-and-peer-review-process%23loc-peer-review-history&data=04%7C01%7CJ.Whiting2%40exeter.ac.uk%7Cbf71176471e144b229c708d8b640947f%7C912a5d77fb984eeeaf321334d8f04a53%7C0%7C0%7C637459735781224594%7CUnknown%7CTWFpbGZsb3d8eyJWIjoiMC4wLjAwMDAiLCJQIjoiV2luMzIiLCJBTiI6Ik1haWwiLCJXVCI6Mn0%3D%7C1000&sdata=Xe0q7x7Htc0u99UW00XGfA0wdkpb8M9IOE5YoHmUhEA%3D&reserved=0)). If published, this will include your full peer review and any attached files.

If you choose “no”, your identity will remain anonymous but your review may still be made public.

**Do you want your identity to be public for this peer review?** For information about this choice, including consent withdrawal, please see our [Privacy Policy](https://eur03.safelinks.protection.outlook.com/?url=https%3A%2F%2Fwww.plos.org%2Fprivacy-policy&data=04%7C01%7CJ.Whiting2%40exeter.ac.uk%7Cbf71176471e144b229c708d8b640947f%7C912a5d77fb984eeeaf321334d8f04a53%7C0%7C0%7C637459735781234589%7CUnknown%7CTWFpbGZsb3d8eyJWIjoiMC4wLjAwMDAiLCJQIjoiV2luMzIiLCJBTiI6Ik1haWwiLCJXVCI6Mn0%3D%7C1000&sdata=6fJC7zJ8rVHB8v2vnzWy3y1xZ5ylmic55oLTNF2%2FZc8%3D&reserved=0).

Reviewer #1: No

Reviewer #2: No

Reviewer #3: No
